# Review of Australasian spider flies (Diptera, Acroceridae) with a revision of *Panops* Lamarck

**DOI:** 10.3897/zookeys.172.1889

**Published:** 2012-03-01

**Authors:** Shaun L. Winterton

**Affiliations:** 1 California State Collection of Arthropods, California Department of Food & Agriculture, Sacramento, California, USA

**Keywords:** cybertaxonomy, spider parasitoid

## Abstract

The Australasian spider flies (Diptera: Acroceridae) are reviewed, with all eight currently recognized genera diagnosed and figured. The panopine genus *Panops* Lamarck, 1804 from Australia and Indonesia is revised with four new species described, increasing the total number of species in the genus to nine: *Panops aurum* **sp. n.**, *Panops danielsi* **sp. n.**, *Panops jade* **sp. n.** and *Panops schlingeri* **sp. n.** Five species of *Panops* are redescribed: *Panops austrae* Neboiss, 1971, *Panops baudini* Lamarck, 1804, *Panops boharti* (Schlinger, 1959), **comb. n.**, *Panops conspicuus* (Brunetti, 1926) and *Panops grossi* (Neboiss, 1971), **comb. n.** The monotypic genera *Neopanops* Schlinger, 1959 and *Panocalda* Neboiss, 1971 are synonymized with *Panops*. Keys to genera of Australasian Acroceridae and species of *Panops*, *Helle* Osten Sacken, 1896 and Australasian *Pterodontia* Gray, 1832 are included.

## Introduction

**Figure 1.**
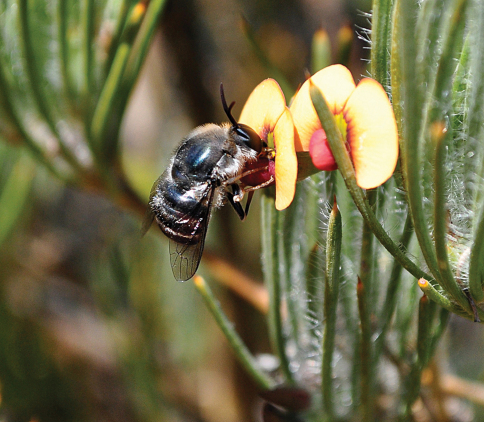
*Panops baudini* Lamarck feeding on *Daviesia croniniana* F.Muell. (Fabaceae), photographed during September in Boorabbin National Park, Western Australia. Photograph by Dan Schoknecht (Western Australian Museum).

Spider flies (also known as small-headed flies) (Diptera: Acroceridae) are a distinctive group of lower brachyceran flies characterized by unusual adult body shape and highly specialized larval biology as parasitoids of spiders. Adults are recognized as important pollinators of angiosperms (Fig. 1), frequently as strong fliers with greatly elongate mouthparts for feeding in long corolla flowers, although some species have reduced or even vestigial mouthparts`(Schlinger 1981, 1987). Acroceridae comprise approximately 520 species in 53 genera (Pape and Thompson 2010; Gillung and Winterton 2011) occupying most biogeographic regions. The family is presently classified into three extant subfamilies: Acrocerinae, Panopinae and Philopotinae (Schlinger 1981), although recent phylogenetic analyses using DNA sequence data suggest that Acrocerinae are polyphyletic and membership of that subfamily should be re-examined (Winterton et al. 2007). Larvae of Acroceridae are internal parasitoids of juvenile spiders, living internally within the opithsoma of the spider where they attach to the book-lungs of the host via their posterior spiracles. Upon completing development the mature, third instar larva emerges from the dead host before pupating (Schlinger 1987). There are exceptions though, with a Chilean species recorded as ectoparasitic on spiders (i.e. *Sphaerops appendiculata* Philippi, 1865 (Acrocerinae)) (Schlinger 1987), whilst Kerr and Winterton (2008) recently questioned the exclusivity of parasitism of spiders, describing a putative acrocerid planidium on an anystinid mite in Baltic Amber.

The Australasian acrocerid fauna comprises all three subfamilies, although represented by relatively few genera. Two acrocerine genera (*Ogcodes* Latreille, 1797 and *Pterodontia* Gray, 1832) are found throughout the region, and are considered cosmopolitan throughout all major biogeographic regions. Philopotinae are represented by an endemic genus in New Zealand (*Helle* Osten Sacken, 1896) (Paramonov 1955) and a recently described genus endemic to New Caledonia (*Schlingeriella* Gillung & Winterton, 2011).

Panopinae are well represented in the Australasian region.Six genera are described previously from New Zealand (*Apsona* Westwood, 1876), Indonesia (*Neopanops* Schlinger, 1959) and Australia (*Panocalda* Neboiss, 1971, *Panops* Lamarck, 1804, *Mesophysa* Macquart, 1838 and *Leucopsina* Westwood, 1876) (Paramonov 1955, 1957; Schlinger 1959; Neboiss 1971). *Pterodontia* has been considered by some authors to be placed in Panopinae based on the presence of tibial spines (Schlinger 1981, 1987, 2009), but most authors place it in Acrocerinae based on wing venation and antennal characteristics (e.g. Neboiss 1971) and molecular data (Winterton et al. 2007). *Panops* is the most species rich genus in the region and is revised herein. Three species were described previously and treated in the most recent revision of the genus by Neboiss (1971): *Panops austrae* Neboiss, 1971, *Panops baudini* Lamarck, 1804, and *Panops conspicuus* (Brunetti, 1926). An additional four species are described herein (*Panops aurum* sp. n., *Panops danielsi* sp. n., *Panops jade* sp. n. and *Panops schlingeri* sp. n.) whilst another two species are moved from other genera (*Panops boharti* (Schlinger, 1959), comb. n. and *Panops grossi* (Neboiss, 1971), comb. n.). Discovery of these new species of *Panops* has expanded the concept of the genus, with various species exhibiting combinations of characteristics previously used to differentiate *Panops* from *Panocalda* and *Neopanops*– specifically length of the mouthparts and presence and distribution of eye pilosity. Consequently, *Neopanops* and *Panocalda* are newly synonymized with *Panops*. All Australasian acrocerid genera are diagnosed and figured. Four genera of Panopinae are now recognized from the Australasian region, *Apsona* (1 sp.), *Mesophysa* (4 spp.), *Panops* (9 spp.) and *Leucopsina* (2 spp.). Keys to genera of Australasian Acroceridae and species of *Panops*, *Helle* and Australasian *Pterodontia* are included.

## Material and methods

Terminology follows McAlpine (1981) and Schlinger (1981). In most acrocerids, two crossveins span the area between the radial and medial sectors. The proximal crossvein is r-m, while the distal crossvein bisecting cell r_4+5_ (between wing veins M_1_ and R_4+5_, or rarely R_5_) is referred to here as 2r-m following Hardy (1946) and Gillung and Winterton (2011). Annotations of collection label data are included where appropriate in brackets. The following collection codens are cited in the text: Australian Museum (AMS), Australian National Insect Collection (ANIC), California Academy of Sciences (CAS), Canadian National Collection of Insects (CNC); Greg Daniels private collection [to be ultimately deposited in the Australian Museum] (GDCB/AMS), Museum National d’Histoire Naturelle (MNHN), National Museum of Victoria (NMV), Oxford University Museum of Natural History (OUMNH), Queensland Museum (QM), Swedish Museum of Natural History (NHRS), South Australian Museum (SAM), The Natural History Museum (BMNH), Western Australian Museum (WAM). Descriptions were constructed using Lucid Builder 3.5, using a matrix database of character states, which were then exported using the natural language function into XML and a text document. Specimen images were taken at different focal points using a digital camera and subsequently combined into a serial montage image using Helicon Focus software. High-resolution digital images were deposited into Morphbank with embedded URL links within the document between descriptions and Morphbank images. All new nomenclatural acts and literature are registered in Zoobank (Pyle and Michel 2008).

## Taxonomy

### Key to genera of Australasian Acroceridae

**Table:** 

1	Postpronotal lobes greatly enlarged, contiguous along midline to form collar for head	Philopotinae, 2
–	Postpronotal lobes not greatly enlarged, widely separate along midline	3
2	Wing with cells d, br, bm, and cu-p present, venation relatively complete (Fig. 3A)	*Helle* Osten Sacken, 1896 (New Zealand)
–	Wing with only cell br present, venation reduced (Fig. 3B)	*Schlingeriella* Gillung & Winterton, 2011 (New Caledonia)
3	Antenna usually styliform or rod-like with multiple terminal setae; wing venation reduced: at most three radial veins present, cells d and basal r_4+5 _merged or absent (Figs 3C, D); tibiae without spines (except *Pterodontia*)	Acrocerinae, 4
–	Antenna with elongate flagellum, cylindrical or flattened, without terminal styliform seta; wing venation complete: four radial veins present, cells d and basal r_4+5 _separate (Figs 2A–D); at least some tibiae with an apical spine on outer margin (absent in *Apsona*)	Panopinae, 5
4	Eye apilose, without setae; venation reduced with many veins absent or poorly defined, almost all cells weakly formed or absent; tibial spines absent (Figs 3C, 63–64)	*Ogcodes* Latreille, 1797 (Cosmopolitan)
–	Eye pilose; all wing veins well defined to wing margin, discal cell and basal portion of r_4+5 _merged into single closed cell; tibial spines present (Figs 3D, 65–66)	*Pterodontia* Gray, 1832 (Cosmopolitan)
5	Eye strongly pilose; antennal flagellum slender and tapered to apex; tibial spines absent (Figs 2A, 4–6)	*Apsona* Westwood, 1876 (New Zealand)
–	Eye apilose or weakly pilose; antennal flagellum thickened to apex; tibial spines present (Australia)	6
6	Eye apilose, or sparsely or partially pilose; wing hyaline; crossvein 2r-m joining to stem R_4+5_ (Fig. 2D)	*Panops* Lamarck, 1804
–	Eye always apilose; wing at least partially infuscate, particularly along anterior margin; crossvein 2r-m joining to vein R_5_ (Fig. 2B–C)	7
8	Dorsal profile of abdomen with swollen, rounded tergites; without transverse yellow band on tergite 3; not wasp-like in appearance (Figs 12–16)	*Mesophysa* Macquart, 1838
–	Dorsal profile of abdomen with truncated tergites raised along posterior margins; transverse yellow band on tergite 3; distinctly wasp-like in appearance (Figs 7–11)	*Leucopsina* Westwood, 1876

**Figure 2.**
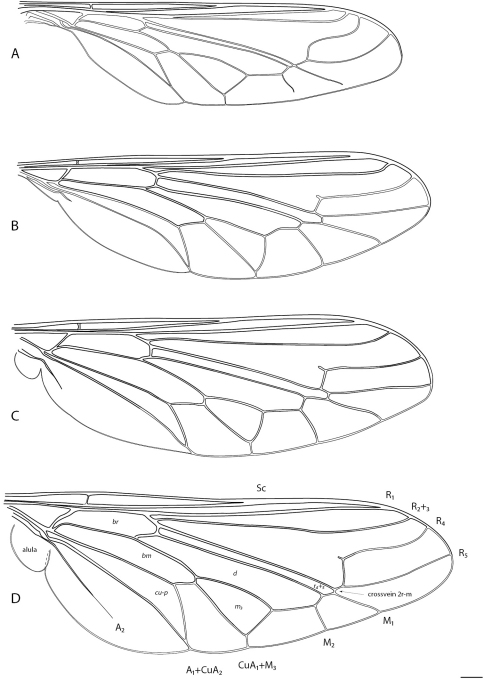
Acroceridae wings. Panopinae: **A** *Apsona muscaria* Westwood **B** *Leucopsina odyneroides* Westwood **C** *Mesophysa tenaria* Neboiss **D** *Panops jade* sp. n. Scale line = 0.2 mm.

**Figure 3.**
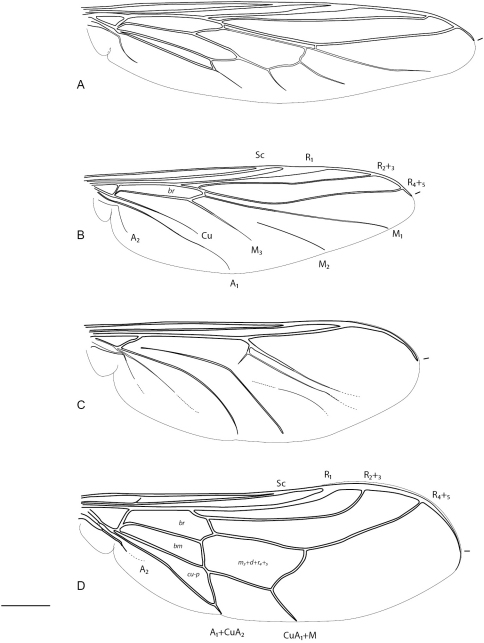
Acroceridae wings. Philopotinae: **A** *Helle rufescens* Brunetti **B** *Schlingeriella irwini* Gillung & Winterton. Acrocerinae **C** *Ogcodes basalis* Walker **D** *Pterodontia davisi* Paramonov (female). Scale line = 0.2 mm.

### Subfamily Panopinae

Schiner, 1868

http://species-id.net/wiki/Panopinae

#### Type genus.

*Panops* Lamarck, 1804: 263.

#### Diagnosis.

Usually large and densely pilose, body shape never arched; antennal flagellum elongate cylindrical to paddle-shaped, sometimes tapered but never stylate, usually lacking terminal setae; postpronotal lobes never meeting medially; wing venation complete to wing margin (rarely reduced), cells m_3_, d, bm and basal r_4+5 _typically present, closed distally; tibial spines present (rarely absent); larvae exclusively parasitoids of mygalomorph spiders.

### Australasian genera

*Apsona* Westwood, 1876; *Leucopsina* Westwood, 1876; *Mesophysa* Macquart, 1838; *Panops* Lamarck, 1804.

#### Apsona

Westwood, 1876

http://species-id.net/wiki/Apsona

Figs 2A 4 [Fig F5] –6

Apsona Westwood, 1876: 510 – Bigot 1890: 317; Hutton 1901: 27; Paramonov 1955: 19; Schlinger 1966: 112; Schlinger and Jefferies 1989: 375. Type species: *Apsona muscaria* Westwood, 1876 by monotypy.

##### Diagnosis.

 Body length: 7–9 mm. Colouration metallic green; head width slightly smaller than thorax width, hemispherical; postocular ridge and occiput rounded; three ocelli; posterior margin of eye rounded; eye pilose (dense); position of antenna on frons nearer to ocellar tubercle; eyes contiguous above and below antennal base; palpus present; proboscis longer than head length; flagellum shape elongate, tapered apically, apex lacking terminal setae; scapes separate; subscutellum not enlarged, barely visible; tibial spines absent; pulvilli present; wing hyaline, markings absent; costa circumambient, costal margin straight apically in both sexes; humeral crossvein present; radial veins curved towards wing anterior margin; R_1_ not inflated distally; pterostigma and cell r_1_ membranous, not ribbed; R_2+3 _present; R_4+5 _present as forked petiolate veins; cell r_4+5 _bisected by 2r-m, basal cell narrow elongate, closed; 2r-m very short, joining M_1_ to stem R_4+5_; R_4 _without spur vein; medial vein compliment with M_1_, M_2_ and M_3_ present (M_3_ fused with CuA_1_); discal cell closed completely; M_1_ and M_2_ usually not reaching wing margin; cell m_3_ present; CuA_1_ joining M_3_, petiolate to wing margin; CuA_2_ fused to A_1_ before wing margin, petiolate; wing microtrichia absent; anal lobe well developed; alula absent; abdominal tergites smooth, rounded; abdomen shape greatly rounded, inflated, conical posteriorly.

**Figure 4.**
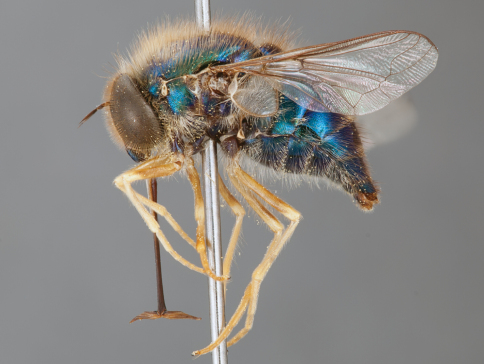
*Apsona muscaria* Westwood, male, lateral view [700415]. Body length = 8.0 mm.

**Figure 5. F5:**
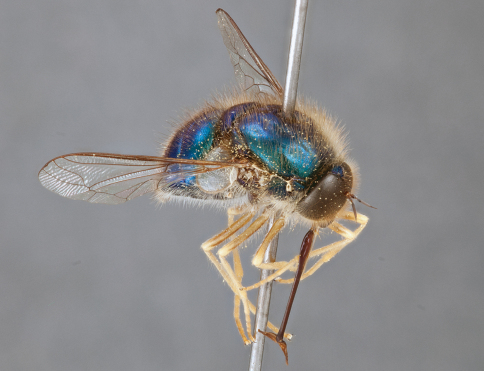
*Apsona muscaria* Westwood, male, oblique view [700418]. Body length = 8.0 mm.

**Figure 6.**
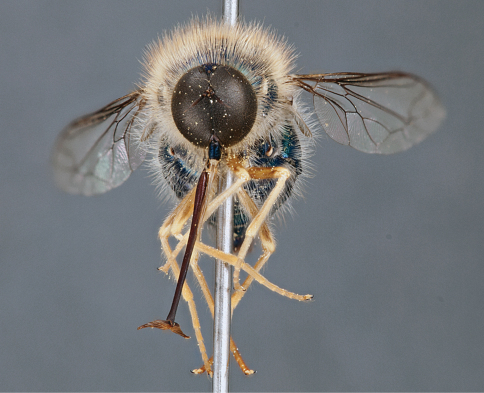
*Apsona muscaria* Westwood, male, anterior view [700419]. Body length = 8.0 mm.

##### Included species.

*Apsona muscaria* Westwood, 1876.

##### Comments.

*Apsona* is a monotypic genus endemic to New Zealand and can be readily differentiated from all other Panopinae based on the lack of tibial spines. *Apsona* shows little relationship to the rest of the Australasian Panopinae and shows remarkable similarity to the New World genus *Eulonchus* Gerstaecker, 1856, sharing numerous characteristics such as metallic green colouration, antennal shape, dense eye pilosity, elongate mouthparts, eyes contiguous below antennal base and absence of an alula (Paramonov 1955).

#### Leucopsina

Westwood, 1876

http://species-id.net/wiki/Leucopsina

Figs 2B 7 [Fig F8] [Fig F9] [Fig F10] –11

Leucopsina Westwood, 1876: 510 – Bigot 1890: 314, 315; Hardy 1921: 78; Paramonov 1957: 524; Neboiss 1971: 219; Schlinger and Jefferies 1989: 375. Type species: *Leucopsina odyneroides* Westwood, 1876 by monotypy.

##### Diagnosis.

Body length: 9.0 mm [male], 12.0 mm [female]. Colouration black and yellow [wasp mimic]; head slightly smaller than thorax width, shape hemispherical; postocular ridge and occiput rounded; three ocelli, anterior ocellus reduced in size (female) or absent (male); posterior margin of eye emarginate; eye apilose; position of antennae on head adjacent to ocellar tubercle; male frons width above antennal base not contiguous, eyes contiguous below antennal base; palpus present; proboscis greater than head length; flagellum shape elongate, cylindrical; apex lacking terminal setae; scapes separate; subscutellum enlarged; tibial spines present; pulvilli present; wing markings present (infuscate anteriorly); costa circumambient (weaker along anal margin); costal margin straight; humeral crossvein present; radial veins straight; R_1_ not inflated distally; pterostigma and cell r_1_ membranous, not ribbed; R_2+3 _present; R_4+5 _originating separately from cell r_4+5 _(or at same point); cell r_4+5 _bisected by 2r-m, basal cell narrow elongate, closed; 2r-m joining M_1_ to R_5_; R_4_ with spur vein; medial vein compliment: M_1_, M_2_ and M_3 _present (M_3_ fused with CuA_1_); discal cell closed completely; medial veins reaching wing margin; cell m_3_ present; CuA_1_ joining M_3, _petiolate to margin; CuA_2_ fused to A_1_ before wing margin, petiolate; wing microtrichia absent; anal lobe well-developed; alula weakly developed; abdominal tergites smooth, rounded, tergites raised along posterior margins; abdomen constricted anteriorly.

**Figure 7.**
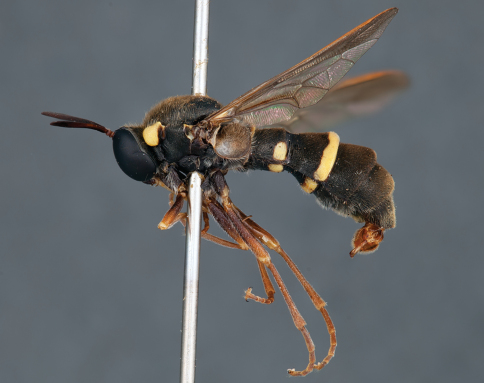
*Leucopsina odyneroides* Westwood, male, lateral view [700421]. Body length = 9.0 mm.

**Figure 8. F8:**
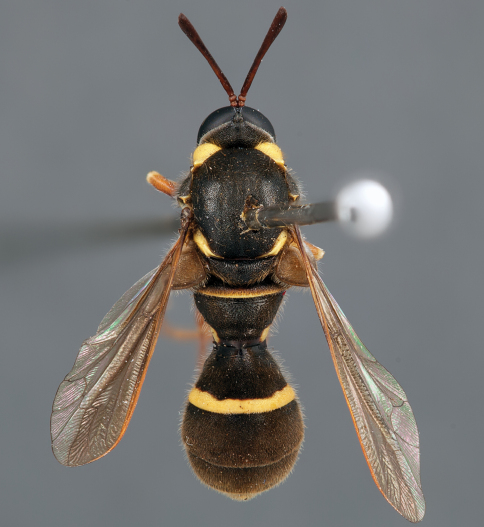
*Leucopsina odyneroides* Westwood, male, dorsal view [700423]. Body length = 9.0 mm.

**Figure 9. F9:**
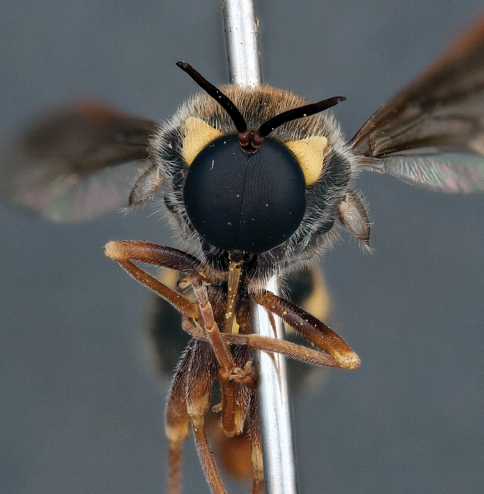
*Leucopsina odyneroides* Westwood, male, anterior view [700426]. Body length = 9.0 mm.

**Figure 10. F10:**
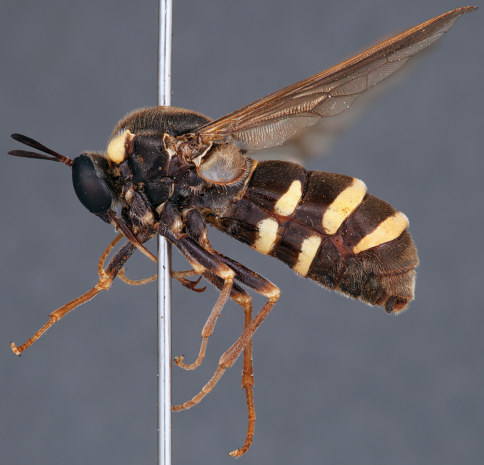
*Leucopsina odyneroides* Westwood, female, lateral view [700436]. Body length = 12.0 mm.

**Figure 11.**
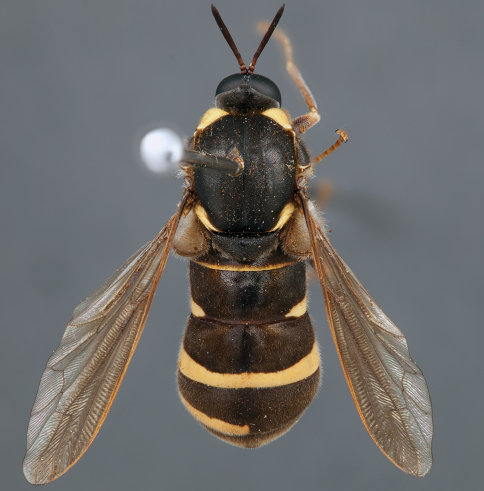
*Leucopsina odyneroides* Westwood, female, dorsal view [700447]. Body length = 12.0 mm.

##### Included species.

*Leucopsina burnsi* (Paramonov, 1957); *Leucopsina odyneroides* Westwood, 1876.

##### Comments.

*Leucopsina* is an endemic Australian genus of contrastingly coloured yellow and black flies, with distinct sexual dimorphism between males and females; male having more pronounced constriction of the abdomen anteriorly. The body colouration, darkening of the costal wing margin and abdominal waist allows members of this genus to be convincing wasp mimics (Neboiss 1971). *Leucopsina* can be differentiated from all other acrocerid genera by the wasp mimicking habitus, elongate cylindrical flagellum, apilose eyes and elongate mouthparts. Neboiss (1971) provides a key to species of this genus. *Leucopsina burnsi* was originally described as a variety of *Panops flavipes* (=*Mesophysa flavipes* Latreille, 1811) but subsequently transferred to *Leucopsina* and thoroughly differentiated from *Leucopsina odyneroides* by Neboiss (1971).

#### Mesophysa

Macquart

http://species-id.net/wiki/Mesophysa

Figs 2C 12 [Fig F13] [Fig F14] [Fig F15] –16

Mesophysa Macquart, 1838: 166 – Blanchard 1840: 584; Westwood 1876: 517; Brunetti 1926: 580; Edwards 1930: 193; Neboiss 1971: 214; Schlinger and Jefferies 1989: 376. Type species: *Mesophysa scapularis* Macquart, 1838 by subsequent designation of Brunetti 1926: 580 [= *Panops flavipes* Latreille, 1811].

##### Diagnosis.

Body length: 8.0–10.0 mm [male], 9.0–11 mm [female]. Colouration non-metallic, usually matte greenish hue; head size slightly smaller than thorax width; shape hemispherical; postocular ridge and occiput rounded; three ocelli; posterior margin of eye emarginate; eye apilose; antennae positioned on head adjacent to ocellar tubercle; eyes not contiguous above antennal base, contiguous below antennal base; palpus present; proboscis greater than head length; flagellum shape elongate, cylindrical (flattened), truncated apically [more pronounced in male]; scapes separate; flagellum apex lacking terminal setae; subscutellum not enlarged, barely visible; tibial spines present; pulvilli present; wing infuscate, markings present; costa circumambient (weaker along anal margin); costal margin straight apically; humeral crossvein present; radial veins straight; R_1_ not inflated distally; pterostigma and cell r_1_ membranous, not ribbed; R_2+3 _present; R_4+5 _originating separately from cell r_4+5_; cell r_4+5 _bisected by 2r-m, basal cell narrow elongate, closed; 2r-m, joining M_1_ to R_5_; R_4_ with spur vein; medial vein compliment with M_1_, M_2_ and M_3_ present (M_3_ fused with CuA_1_); discal cell closed completely; medial veins reaching wing margin; cell m_3_ present; CuA_1_ joining M_3_, petiolate to margin; CuA_2_ fused to A_1_ before wing margin, petiolate to margin; wing microtrichia absent; anal lobe well developed; alula well developed; abdominal tergites smooth, rounded; abdomen shape rounded, cylindrical, similar width to thorax or constricted anteriorly (male), tergites raised along posterior margins.

**Figure 12.**
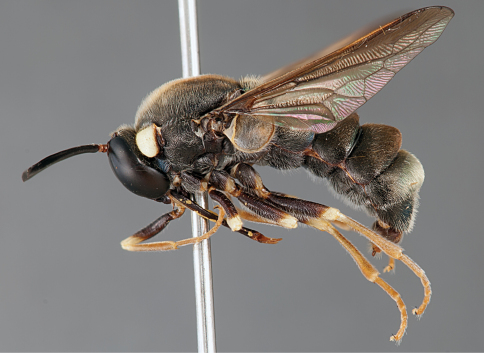
*Mesophysa tenaria* Neboiss, male, lateral view [700448]. Body length = 10.0 mm.

**Figure 13. F13:**
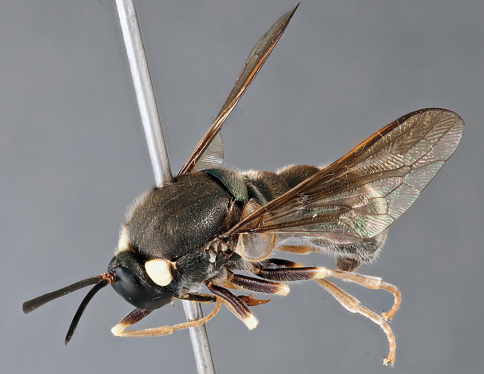
*Mesophysa tenaria* Neboiss, male, oblique view [700450]. Body length = 10.0 mm.

**Figure 14. F14:**
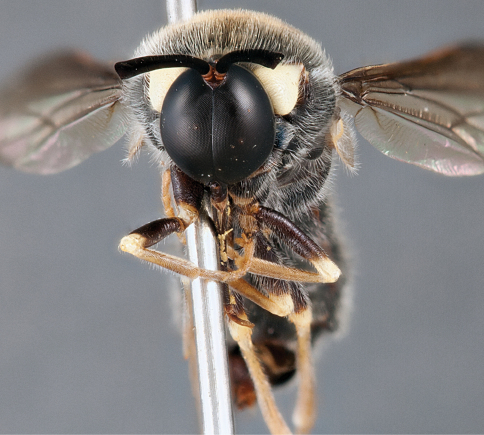
*Mesophysa tenaria* Neboiss, male, anterior view [700452]. Body length = 10.0 mm.

**Figure 15. F15:**
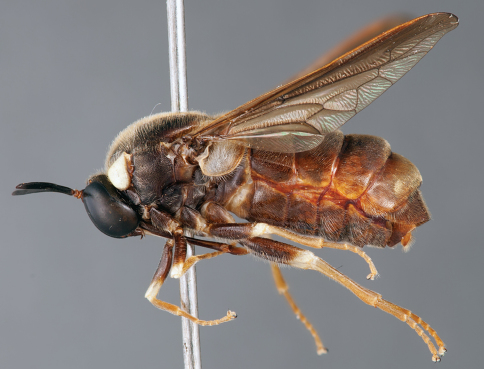
*Mesophysa tenaria* Neboiss, female, lateral view [700453]. Body length = 11.0 mm.

**Figure 16.**
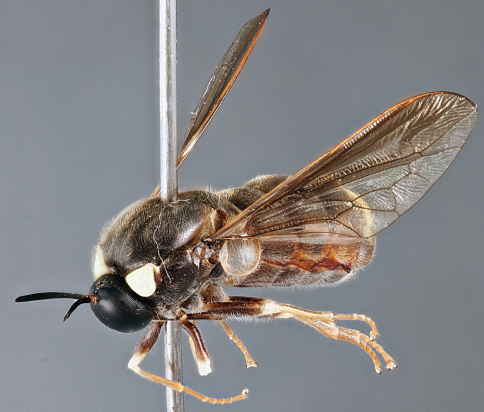
*Mesophysa tenaria* Neboiss, female, oblique view [700454]. Body length = 11.0 mm.

##### Included species.

*Mesophysa flavipes* (Latreille, 1811); *Mesophysa ilzei* Neboiss, 1971; *Mesophysa tenaria* Neboiss, 1971; *Mesophysa ultima* Neboiss, 1971.

##### Comments.

*Mesophysa* is an endemic eastern Australian genus closely related to *Leucopsina*. They share a similar habitus with narrowing of the abdomen anteriorly (more pronounced in *Leucopsina*), apilose eyes, infuscate wings and flagellum shape, as well as the crossvein 2r-m joining to R_5 _rather than to the stem R_4+5_. This genus can be differentiated from *Leucopsina* by the lack of black and yellow markings. *Mesophysa* has been considered a synonym of *Panops* by some authors (Erichson 1840; Kertész 1909; Edwards 1930; Hardy 1946; Paramonov 1957) and treated as separate genera by others (e.g. Brunetti 1926; Neboiss 1971). This was complicated by an incorrect synonymy of *Panops* with the distantly related South American genus *Lasia* Wiedemann, 1824 by Kertész (1909) (see discussion in Neboiss 1971). Neboiss (1971) provides a key to species of this genus.

#### Panops

Lamarck, 1804

http://species-id.net/wiki/Panops

Figs 1 2D 17 [Fig F18] [Fig F19] [Fig F20] [Fig F21] [Fig F22] [Fig F23] [Fig F24] [Fig F25] [Fig F26] [Fig F27] [Fig F28] [Fig F29] [Fig F30] [Fig F31] [Fig F32] [Fig F33] [Fig F34] [Fig F35] [Fig F36] [Fig F37] [Fig F38] [Fig F39] [Fig F40] [Fig F41] [Fig F42] [Fig F43] [Fig F44] [Fig F45] [Fig F46] [Fig F47] [Fig F47] [Fig F48] [Fig F49] [Fig F50] [Fig F51] [Fig F52] [Fig F53] [Fig F54] –55

Panops Lamarck, 1804: 263 – Latreille 1804: 191, 1809: 316, 1810: 392, 443, 1811: 707, 1816: 608, 1825: 492, 1829: 461; Lamarck 1812: 56; Wiedemann 1830: 18; Macquart 1838: 166; Blanchard 1840: 583; Erichson 1840: 140; Walker 1855: 332; Schiner 1868: 140; Westwood 1876: 509; Bigot 1890: 314; Hardy 1921: 76, 1946: 66; Brunetti 1926: 580; Paramonov 1957: 525; Neboiss 1971: 208; Schlinger and Jefferies 1989: 376. Type species: *Panops baudini* Lamarck, 1804 by monotypy.
Epicerina Macquart, 1850: 97 – Bigot 1890: 316. Synonymy in: Hardy 1921: 79; Hardy 1946: 66; Paramonov 1957: 521. Type species: *Epicerina nigricornis* Macquart, 1850 by original designation.
Neopanops Schlinger, 1959: 157 – Schlinger and Jefferies 1989: 376. Type species: *Neopanops boharti*, Schlinger, 1959 by original designation. syn. n.
Panocalda Neboiss, 1971: 212 – Schlinger and Jefferies 1989: 376. Type species: *Panocalda grossi*, Neboiss, 1971 by original designation. syn. n.

##### Diagnosis.

**Figure 17.**
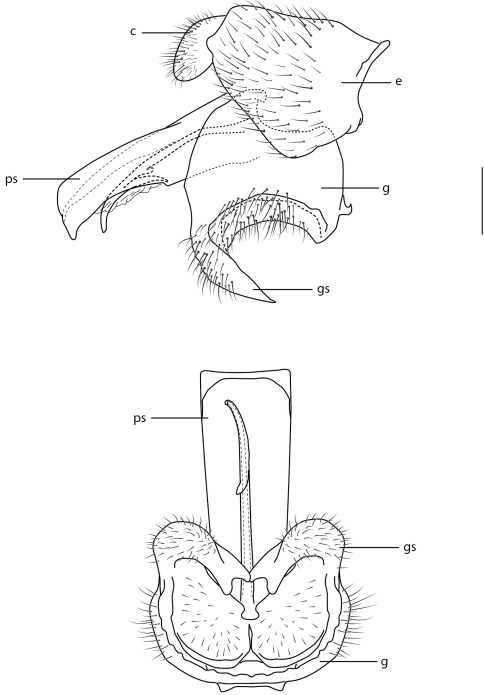
*Panops baudini* Lamarck. **A** male genitalia, lateral view **B** same, ventral view. Scale line = 0.2 mm. Abbreviations: **c** cercus; **e** epandrium; **g** gonocoxite; **gs** gonostylus; **ps** parameral sheath of aedeagus.

 Body length: 8.0–12.5 mm [male], 9.5–14.5 mm [female]. Colouration non-metallic or metallic; head slightly smaller than thorax width, shape hemispherical; postocular ridge and occiput rounded; three ocelli, anterior ocellus reduced in size or absent; posterior margin of eye emarginate; eye apilose or pilose (sparse) (sometimes localized dorsally); position of antennae on head adjacent to ocellar tubercle; eyes not contiguous above antennal base, contiguous below antennal base; palpus present; proboscis length variable, less than or greater than head length; flagellum shape elongate, slightly tapered (female) or elongate, cylindrical (male); flagellum apex lacking terminal setae; scapes separate; subscutellum not enlarged, barely visible; tibial spines present; pulvilli present; wing hyaline, markings absent; costa circumambient (weaker along anal margin); costal margin at pterostigma straight; humeral crossvein present; R_1_ not inflated distally; pterostigma and cell r_1_ membranous, not ribbed; vein R_2+3 _present; R_4_ and R_5_ present as forked petiolate veins; radial veins straight towards wing apex, slightly angled anteriorly; cell r_4+5 _bisected by 2r-m, basal cell narrow elongate, closed; 2r-m joining M_1_ to stem R_4+5_; R_4 _with or without spur vein; medial vein compliment with M_1_, M_2_ and M_3_ present; discal cell closed completely; medial veins reaching wing margin; cell m_3_ present; CuA_1_ joining M_3_, petiolate to wing margin; CuA_2_ fused to A_1_ before wing margin, petiolate to margin; wing microtrichia absent; anal lobe well developed; alula well developed; abdominal tergites smooth, rounded; abdomen shape greatly rounded, inflated (larger in female). Male genitalia (Fig. 17) typical for Panopinae and varying little between species: gonostylus fused with gonocoxite and non-articulated, but with lightly sclerotized areas ventrally indicating flexion of gonostylus with gonocoxite; gonostylus as ventrally curved process with cup-like ventromedial surface; aedeagus consisting of flattened quadrangular, or cylindrical, parameral sheath with ventral rod-like structure with apical gonopore; ejaculatory apodeme poorly developed.

##### Included species.

*Panops aurum* sp. n.; *Panops austrae* Neboiss, 1971; *Panops baudini* Lamarck, 1804; *Panops boharti* (Schlinger, 1959) comb. n.; *Panops conspicuus* (Brunetti, 1926); *Panops danielsi* sp. n.; *Panops grossi* (Neboiss 1971) comb. n.; *Panops jade* sp. n.; *Panops schlingeri* sp. n.

##### Comments.

*Panops* is the type genus for the subfamily Panopinae and includes some large metallic coloured species. The genus is endemic to Australia and neighbouring Papua region of Indonesia. The original concept of the genus was expanded to include species from the New World by some authors, but these have subsequently been placed in the separate and distantly related genus *Lasia* Wiedemann, 1824 (e.g. *Lasia metallica* Rondani, 1863; *Lasia ocelliger* (Wiedemann, 1830)). Bequaert (1931) and later Neboiss (1971), discuss the historically confused and intertwined generic concepts of *Lasia* and *Panops* (sometimes including *Mesophysa*) in previous treatments of the group by various authors. Based on a series of characters, it is clear that those Australasian species are placed in *Panops* or *Mesophysa*, while the New World species are placed in *Lasia*. In his description of *Neopanops*, Schlinger (1959) suggested that the genus was closely related to *Panops* and provided an extensive list of characters distinguishing the two. Similarly, Neboiss (1971) provided a list of characteristics to differentiate *Panocalda* from the closely related *Panops* and *Neopanops*. Both Schlinger (1959) and Neboiss (1971) distinguished their respective genera based on characters such as eye pilosity, length of proboscis, shape of ocellar tubercle, palpi length, head width, parafacial pilosity and wing length. With the inclusion of the four new species described here, and a critical re-examination of the characters used to differentiate *Neopanops* and *Panocalda* from *Panops*, it is clear that all of these characters are variable and that only one genus is warranted. Some species of *Panops* have pilose eyes, either uniformly sparse and minute (i.e. *Panops danielsi* sp. n., *Panops boharti* comb. n., *Panops baudini*) or localized (*Panops grossi* comb. n.), with the other species being apilose. In no species of *Panops* are the eyes uniformly dense pilose, as is found in most other panopine genera (e.g. *Apsona*, *Lasia*). This paucity of eye pilosity is shared with only a few other genera, including the Australian *Leucopsina* and *Mesophysa*, as well as the highly derived genus *Corononcodes* Speiser, 1920 from the Palaearctic and Afrotropical regions. Proboscis length is a frequently used character in acrocerid taxonomy, but in *Panops* the length is dramatically variable, with a proboscis much shorter than the head height in some species (e.g. *Panops jade* sp. n., *Panops schlingeri* sp. n., *Panops boharti* comb. n.) while the rest have a proboscis longer than the head height. *Panops* is a variable genus, but can be differentiated from all other Panopinae based on the diagnosis above, and specifically from all other genera in the Australasian region based on tibial spines being present (*cf.* *Apsona*) and wing crossvein 2r-m joining to R_4+5 _(*cf.* *Leucopsina*, *Mesophysa*). Like most acrocerids, species of *Panops* display distinct sexual dimorphism with males often have slightly smaller body size and larger antennae than females. Many Old World panopine genera (e.g. *Apsona*, *Panops*, *Rhysogaster* Aldrich, 1927) have a distinctive unidirectional arrangement of the pile on the head and thorax, giving the individual a dramatic change in appearance when viewed head on (e.g. Figs 20, 23, 40); the biological significance of this is unknown.

##### Key to *Panops* species.

*Panops baudini* keys to two couplets as the eye pilosity is extremely minute in some individuals and may be overlooked. Females are unknown for *Panops boharti* comb. n. and *Panops aurum* sp. n., whilst males are unknown for *Panops schlingeri* sp. n.

**Table:** 

1	Eye sparsely pilose (Fig. 39) or pilosity localized dorsally (Fig. 44)	2
–	Eye completely apilose (Fig. 18)	5
2	Proboscis elongate, length greater than head height (Fig. 18)	3
–	Proboscis very short, hardly projecting from oral cavity, shorter than head height (Figs 44, 50)	4
3	Postpronotal lobe dark, concolourous with rest of pleuron (widely distributed in Australia) (Figs 25–30)	*Panops baudini* Lamarck,1804
–	Postpronotal lobe yellow, pleuron greenish (Queensland) (Figs 39–43)	*Panops danielsi* sp. n.
4	Eye extending posteriorly beyond widest part of head; eye with sparse, minute pile of uniform length across eye (length subequal to width of lateral ocellus); ocellar tubercle not touching margin of eye; palpus as long or longer than proboscis (Papua) (Figs 31–33)	*Panops boharti* (Schlinger, 1959), comb. n.
–	Eye not extending posteriorly beyond widest part of head; eye pilose on dorsal-lateral region only, pile denser and more elongate (length much greater than width of lateral ocellus); ocellar tubercle touching margin of eye; palpus half as long as proboscis (South Australia) (Figs 44–47)	*Panops grossi* (Neboiss, 1971), comb. n.
5	Proboscis short, hardly projecting from oral cavity	6
–	Proboscis elongate, length equal to, or greater than head height	7
6	Postpronotal lobes dark yellow; femora dark brown, rest of legs cream (Northern Territory) (Figs 53–55)	*Panops schlingeri* sp. n.
–	Postpronotal lobes and legs dark, concolourous with rest of body (Queensland) (Figs 48–52)	*Panops jade* sp. n.
7	Postpronotal lobes pale, contrasting with rest of thorax (Figs 34–38)	*Panops conspicuus* (Brunetti, 1926)
–	Postpronotal lobes dark, concolourous with rest of thorax (Figs 21, 28)	8
8	Body metallic, thorax green, abdomen violet; margin of lower calypter relatively dark (Figs 21–24) (Western Australia)	*Panops austrae* Neboiss, 1971
–	Thorax mostly glossy black, abdomen often with extensive red-brown to purple laterally; margin of lower calypter relatively pale (Figs 19, 26)	9
9	Face above clypeus apilose; body covered with white setal pile; male distiphallus broad, spatulate (widely distributed in Australia) (Figs 25–30)	*Panops baudini* Lamarck, 1804
–	Face above clypeus with gold setal fringe; body covered with yellow-gold setal pile; male distiphallus narrow (Figs 18–20) (Western Australia)	*Panops aurum* sp. n.

##### Panops aurum sp. n.

urn:lsid:zoobank.org:act:3864CACB-368C-4770-88E8-8346544EBED7

http://species-id.net/wiki/Panops_aurum

**Figure 18. F18:**
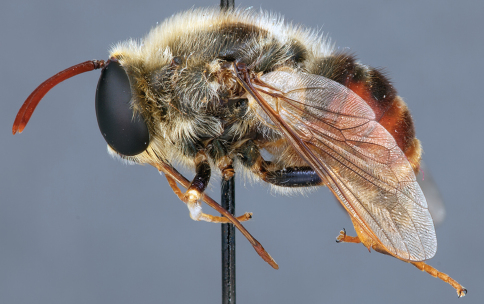
*Panops aurum* sp. n., male, lateral view [700495]. Body length = 11.0 mm.

Figs 18 [Fig F19] –20

###### Type material. 

**Holotype** male, AUSTRALIA: **Western Australia:** Darlington, 450 ft., E.S. Ross, D.Q. Cavagnaro, 5.ix.1962 [-31.901, 116.081] (CAS).

###### Diagnosis.

Eye apilose; proboscis longer than head height; body non-metallic; antennae red-brown; parafacial with yellow marginal pile; postpronotal lobe concolourous with rest of thorax; legs dark yellow, femora brown-black.

###### Description. 

Body length: 11.0 mm (male). Headwitheye apilose; ocellar tubercle raised laterally; medial ocellus absent; occiput brown-black, occipital pile yellow, postocular ridge and gena overlain with grey pubescence; clypeus length equal to oral cavity, brown-black; palpus yellow; margin of oral cavity (parafacial) densely pilose (yellow); proboscis longer than head height; flagellum apex of uniform width, truncated apically, flagellum red-brown; scape and pedicel brown. Thorax with postpronotal lobe brown-black; scutum black, scutal vestiture dense yellow-gold pile; scutellum black; pleuron black; coxae black; femora brown-black, apices dark yellow; tibiae dark yellow; tarsi dark yellow; lower calypter white with dark yellow margin; wing hyaline, venation dark; vein R_4_ without spur vein. Abdomen shape rounded globose, much larger than thorax, colour orange-red to yellow, dark markings anteriorly and medially, vestiture dense elongate pile, yellow anteriorly, brown posteriorly on tergites 2–5.

**Figure 19. F19:**
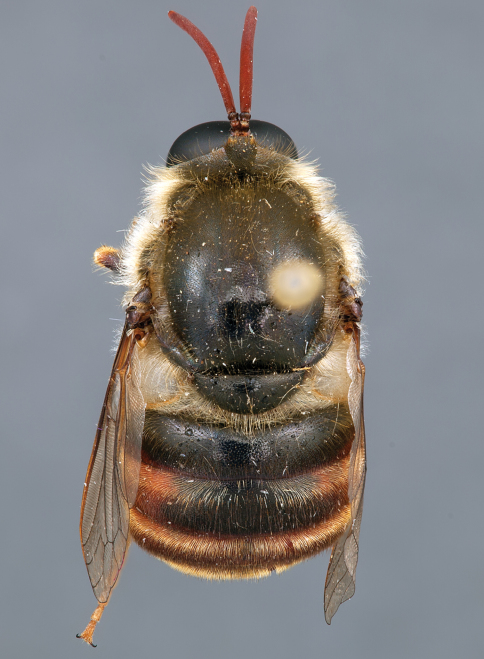
*Panops aurum* sp. n., male, dorsal view [700496]. Body length = 11.0 mm.

**Figure 20. F20:**
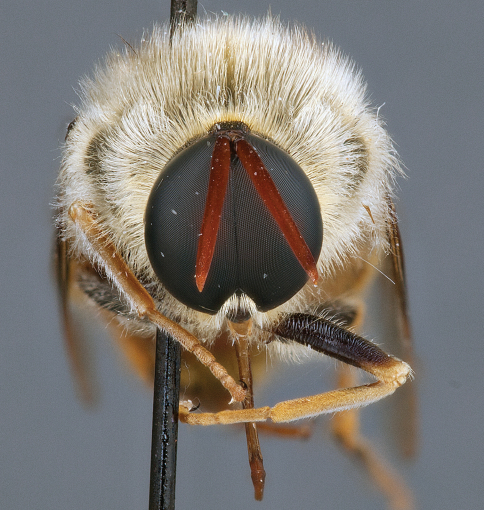
*Panops aurum* sp. n., male, anterior view [700497]. Body length = 11.0 mm.

###### Etymology.

The specific epithet is derived from the Latin, *aurum* – gold; referring to the distinctive golden setal pile on the head and thorax.

###### Comments.

*Panops aurum* sp. n. is known only from a single male specimen from Western Australia. The fringing yellow setae around the oral cavity and yellow pile on the thorax are distinctive for the species.

##### Panops austrae

Neboiss, 1971

http://species-id.net/wiki/Panops_austrae

**Figure 21. F21:**
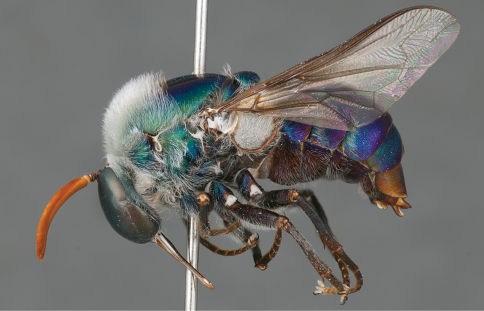
*Panops austrae* Neboiss, male, lateral view (partially denuded) [700499]. Body length = 8.0 mm.

Figs 21 [Fig F22] [Fig F23] –24

Panops austrae Neboiss, 1971: 209 – Schlinger and Jefferies 1989: 376.

###### Type material examined. 

**Holotype** female, AUSTRALIA: **Northern Territory:** nr. Mount Olga [-25.3, 130.73], C.A., Paul Genery, ix.1960, picked up dead in sand (Type- T.4177) (NMV).

###### Other material examined.

AUSTRALIA: **Western Australia:** male, Wialki [-30.483, 118.117], R. P. McMillan, 12.x.1983 (WAM); male, W of Norseman, *Eucalyptus* woodland, dry gully to salt lake, Malaise trap, C. Lambkin et al., ANIC bulk sample 2184, 1-17.xi.2003 271m [-32.186, 121.721] (ANIC).

###### Diagnosis.

Eye apilose; proboscis equal to head height; body metallic green-blue; antennae yellow-brown; parafacial without marginal pile; postpronotal lobe concolourous with rest of thorax; legs black.

###### Redescription.

Body length: 8.0–10.0 mm (male), 14.5 mm (female). Head with eye apilose; ocellar tubercle relatively flat, medial ocellus present; occiput metallic green-blue, occipital pile dense, white; postocular ridge and gena overlain with grey pubescence; clypeus length equal to oral cavity, brown-black; palpus white or black; margin of oral cavity (parafacial) glabrous; proboscis equal or slightly longer than head height; flagellum dark yellow-orange, suffused with brown, apex in male tapered, narrow apically; scape and pedicel brown or dark yellow. Thorax postpronotal lobe green; scutum metallic green or metallic blue, scutal vestiture dense white pile; scutellum metallic blue-green; pleuron metallic green or metallic blue; coxae black with metallic blue iridescence; femora black; tibiae black or brown; tarsi black; lower calypter white, with brown margin; wing hyaline (male) or slightly infuscate (female), venation dark; vein R_4_ with spur vein. Abdomen shape rounded globose, much larger than thorax (female) or rounded to conical, not larger than thorax (male), colour metallic green or metallic blue violet, vestiture as minute setae, dense white-silver elongate setae along anterior margin of tergites 2–5.

**Figure 22. F22:**
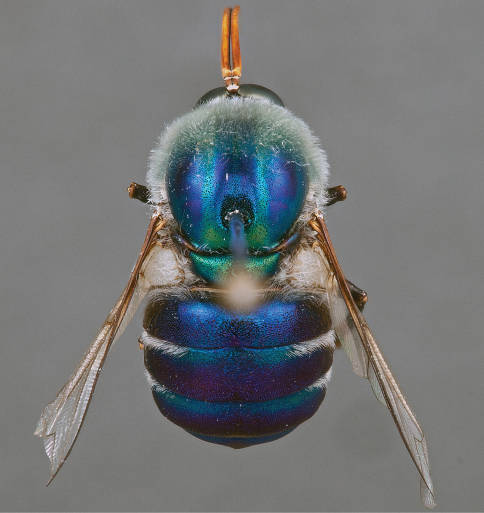
*Panops austrae* Neboiss, male, dorsal view [700502]. Body length = 8.0 mm.

**Figure 23. F23:**
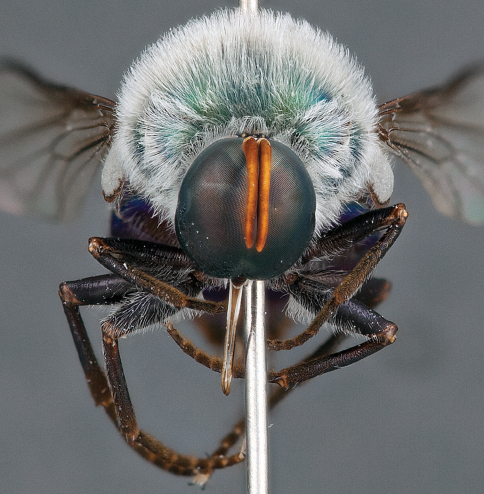
*Panops austrae* Neboiss, male, anterior view [700498]. Body length = 8.0 mm.

**Figure 24. F24:**
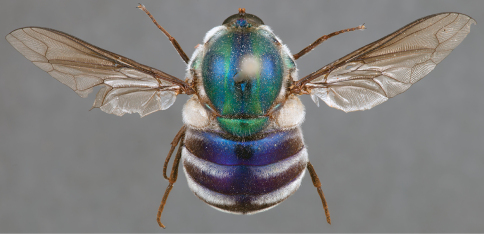
*Panops austrae* Neboiss, female, dorsal view [700508]. Body length = 14.5 mm.

###### Comments.

*Panops austrae* is a large, metallic coloured species similar to *Panops jade* sp. n. and *Panops schlingeri* sp. n. It is easily distinguished from these species by the longer proboscis and dense white thoracic pile. This species is known from remote, arid regions of the Northern Territory and Western Australia.

##### Panops baudini

Lamarck, 1804

http://species-id.net/wiki/Panops_baudini

Figs 1 17 25 [Fig F26] [Fig F27] [Fig F28] [Fig F29] –30

Panops baudini Lamarck, 1804: 265 – Latreille 1809: 316, 1810: 443, 1811: 710; Wiedemann 1830: 19; Erichson 1840: 141; Walker 1855: 333; Kertész 1909: 9; Hardy 1946: 66; Edwards 1930: 193; Paramonov 1957: 526; Neboiss 1971: 208; Schlinger and Jefferies 1989: 376.
Mesophysa marginata Macquart, 1838: 168 – Blanchard 1840: 584.
Epicerina nigricornis Macquart, 1850: 98 – Kertész 1909: 8; Hardy 1918: 61, 1921: 79, 1946: 66; Brunetti 1926: 578.
Panops lamarckianus Westwood, 1876: 508 – Kertész 1909: 9; Paramonov 1957: 526.
Mesophysa australiae Thomson, 1869: 475 – Westwood 1876: 517.
Panops australiae . Kertész, 1909: 8.
Mesophysa baudini Brunetti, 1926: 581.
Panops nigricornis . Hardy, 1946: 66.

###### Type material.

*Panops baudini* Lamarck. **Neotype** female, AUSTRALIA: **New South Wales:** Asquith (nr, Sydney), 10.x.1962, A.L. Dyce (ANIC) (designated by Neboiss 1971). Neboiss (1971) discussed the identity of this species based on the original species description and justification for designating the neotype [examined].

*Mesophysa marginata* Macquart. **Type** female, [no label data] (MHN). See discussion by Neboiss (1971).

*Epicerina nigricornis* Macquart. **Type** male, AUSTRALIA: “2/47 Tasmanie J. Verreaux 1847” (MNHN). See discussion by Paramonov (1957) and Neboiss (1971) regarding synonymy and possible erroneous locality recording.

*Panops lamarckianus* Westwood. **Type** male, AUSTRALIA: **Queensland:** Moreton Bay, 1859 (OUMNH).

*Mesophysa australasiae* Thomson. **Type** male, AUSTRALIA: **New South Wales:** Sydney, Kinb. (NHRS). See discussion by Hardy (1921) and Neboiss (1971) regarding synonymy.

###### Other material examined.

AUSTRALIA: **Queensland:** male, female, Isla Gorge National Park, [-25.183, 149.966] 12.ix.1992, 320m, G. Daniels (GDCB); male, Isla Gorge National Park, [-25.183, 149.966] 11.ix.1992, 320m, R. Eastwood (GDCB); 32 km S Theodore, [-25.166, 150.000], 13.ix.1992, 300m, G. Daniels (GDCB); 2 males, female, 43 km WSW Millmerran, [-27.983, 150.933], 21.ix.1986, G. & A. Daniels (GDCB); 2 females, Lake Broadwater, nr. Dalby, [-27.361, 151.102], site 8, 27.ix.1986, G. & A. Daniels (GDCB); male, Gayndah, Masters (NMV). **New South Wales:** female, Sydney swamps (NMV); male, Sydney, 17.x.1932, G.M. Goldfinch (ANIC); female, Ku-ring-gai Chase National Park [-33.651, 151.201], 2.x.1972, A. & G. Daniels (GDCB); 2 males, Goondera Ridge, Royal National Park [-34.122, 151.063], 24.x.1976, G. & A. Daniels (GDCB). **Victoria:** female, Mitta Mitta River, 8km NW of Dartmouth Dam [-36.566, 147.55], 30.x.1976, A. A. Calder (NMV). **Western Australia:** 3 males, W of Norseman, Eucalyptus woodland, dry gully to salt lake, Malaise trap, C. Lambkin et al., ANIC bulk sample 2184, 1-17.xi.2003 271m [-32.186, 121.721] (ANIC); male, Wongan Hills area [-30.871, 116.771], Greg Guérin, on flowers of *Microcorys* (CAS); female, East Yuna Nature Reserve, 34 km WNW Mullewa [-28.42, 115.42], 23–24.ix.1983, C. & T. Houston, 559-17, on flowers of ?*Helipterum* (WAM); female Australia, Boorabbin Rock National Park [-31.23, 120.16], W Coolgardie, 26.ix.2005, L. Packer (CNC) [not examined but identity confirmed by B. Sinclair].

**Diagnosis.** Eye minutely pilose; proboscis longer than head height; body black (with faint blue iridescence in western population); antennae red-brown to black; parafacial with marginal pile; postpronotal lobe concolourous with rest of thorax; femora black with pale apices, rest of leg dark yellow to white with black on tibiae; abdomen red or yellow laterally; distiphallus broad apically.

###### Redescription.

Body length: 9.5–12.5 mm (male), 11.0–14.0 mm (female). Head with eye sparsely pilose with minute setae (appears apilose); ocellar tubercle raised laterally or relatively flat; medial ocellus reduced; occiput brown-black, occipital pile white, sparse; postocular ridge and gena overlain with grey pubescence; clypeus length equal to oral cavity, brown-black; palpus white or yellow; margin of oral cavity (parafacial) pilose; proboscis longer than head height; flagellum red-brown to black; scape and pedicel brown. Thorax with postpronotal lobe brown-black; scutum black, scutal vestiture dense white pile; scutellum black; pleuron black (thorax with slight bluish iridescence in western populations); coxae black; femora black or brown-black, apices dark yellow; tibiae predominantly black with dark yellow to white (apically); tarsi dark yellow to white; lower calypter white, with yellow margin; wing hyaline (male) or slightly infuscate (female); venation dark; vein R_4_ with spur vein, rarely without. Abdomen shape rounded globose, much larger than thorax, colour highly variable, orange-red to yellow, dark markings anteriorly and medially, or dark yellow, brown anteriorly on tergites 2–6, vestiture as extensive short white-silver pile, longer laterally.

**Figure 25. F25:**
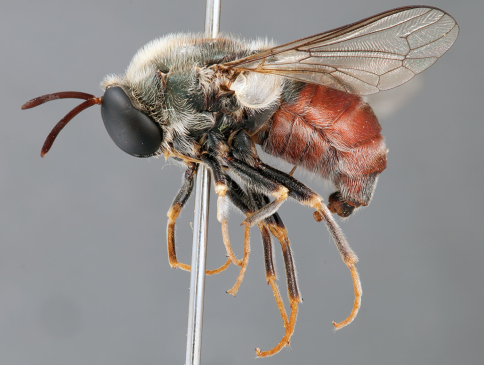
*Panops baudini* Lamarck (western form), male, lateral view [700505]. Body length = 9.5 mm.

**Figure 26. F26:**
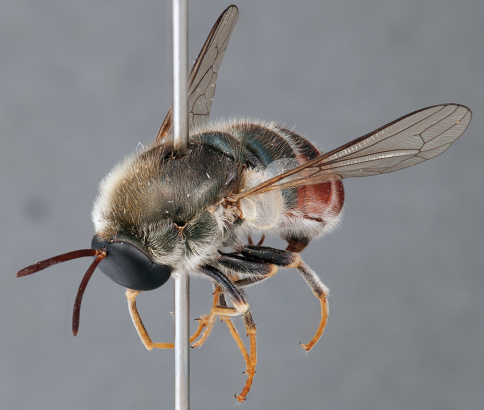
*Panops baudini* Lamarck (western form), male, oblique view [700509]. Body length = 9.5 mm.

**Figure 27. F27:**
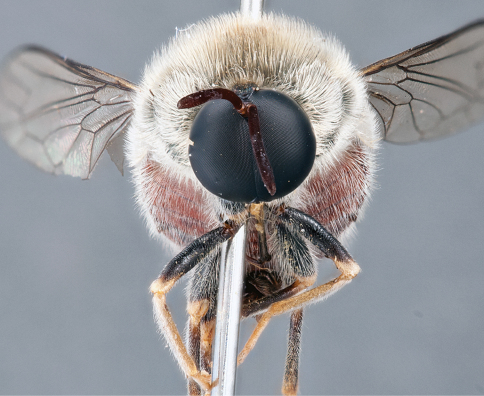
*Panops baudini* Lamarck (western form), male, anterior view [700510]. Body length = 9.5 mm.

**Figure 28. F28:**
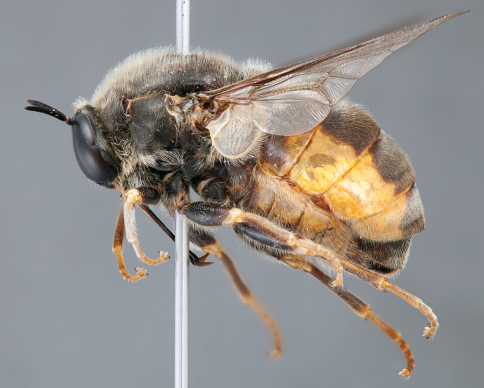
*Panops baudini* Lamarck (eastern form), female, lateral view [700512]. Body length = 12.0 mm.

**Figure 29. F29:**
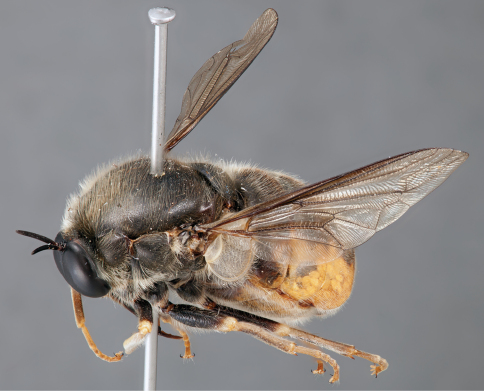
*Panops baudini* Lamarck (eastern form), female, oblique view [700513]. Body length = 12.0 mm.

**Figure 30. F30:**
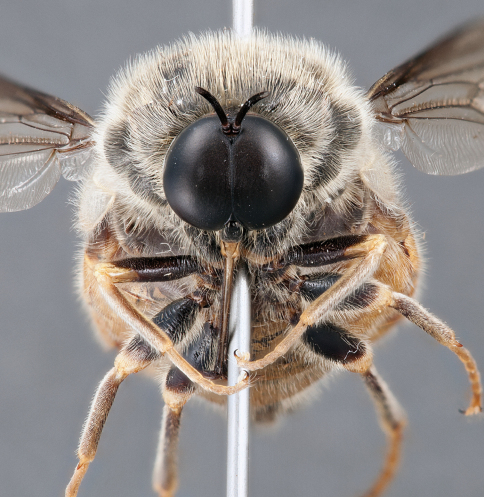
*Panops baudini* Lamarck (eastern form), female, anterior view [700514]. Body length = 12.0 mm.

###### Comments.

The type for the genus, *Panops baudini* is the most commonly represented species in collections. This species is distributed in Queensland, New South Wales, Victoria and Western Australia. The apex of the aedeagus is broad and quadrangular in this species (Fig. 17) while in all other species it is much narrower. The record from Tasmania is apparently erroneous (Neboiss 1971). Western Australian individuals have more reddish colouration laterally on the abdomen, particularly in males, and the body has a bluish iridescence (Fig. 1). This bluish iridescence is not seen in specimens from eastern states.

##### Panops boharti

(Schlinger, 1959) comb. n.

http://species-id.net/wiki/Panops_boharti

**Figure 31. F31:**
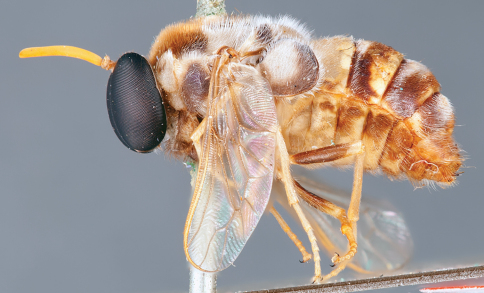
*Panops boharti* (Schlinger) comb. n., male, lateral view [700515]. Body length = 9.0 mm.

Figs 31 [Fig F32] –33

Neopanops boharti Schlinger, 1959: 157 – Neboiss 1971: 212; Schlinger and Jefferies 1989: 376.

###### Type material examined.

**Holotype** male, INDONESIA: **Papua:** Cyclops Mountains, Sabron, 930 ft. [-2.509, 140.523], iv.1936, L. E. Cheesman, B. M. 1936-271 (BMNH).

###### Diagnosis.

Eye pilose; eye extends posteriorly beyond maximum head width; proboscis very short, not extending beyond oral cavity; body brown and yellow; antennae yellow; parafacial without marginal pile; postpronotal lobe cream with brown spot; legs yellow, femora brown with yellow apices; lower calypter cream with brown margin.

###### Redescription.

Body length: 9.0 mm (male). Head with eye sparsely pilose, slightly denser and elongate laterally; eye extends posteriorly beyond maximum head width; ocellar tubercle relatively flat; medial ocellus present; occiput cream, brown suffusion laterally; occipital pile white, sparse; flagellum yellow, apex uniform width, truncated apically; scape and pedicel dark yellow; clypeus minute, yellow-brown; palpus yellow; margin of oral cavity (parafacial) glabrous; proboscis not extending beyond oral cavity. Thorax with postpronotal lobe cream, brown suffusion dorsally; scutum brown, cream posterolaterally; scutal vestiture dense brown and white, matching respective scutal markings; scutellum brown with bluish iridescence, cream laterally; pleuron cream with brown markings; coxae cream with brown markings; femora cream with brown on middle half; tibiae dark yellow; tarsi dark yellow; lower calypter white, brown marginally on membrane; wing hyaline, venation brownish, pale yellow distally along costa and radial veins; vein R_4_ with spur vein. Abdomen rounded globose, slightly larger than thorax, colour dark yellow, brown on tergites 3–6, vestiture minute setae, dense white-silver elongate setae along anterior margin of tergites 2–5.

**Figure 32. F32:**
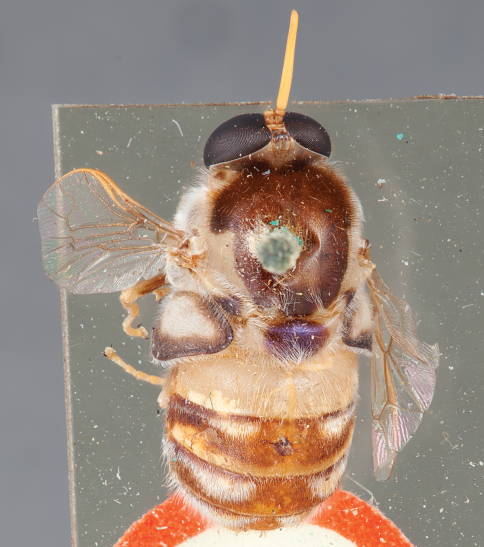
*Panops boharti* (Schlinger) comb. n., male, dorsal view [700517]. Body length = 9.0 mm.

**Figure 33. F33:**
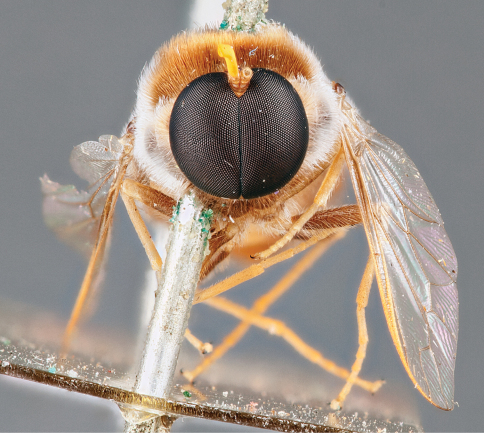
*Panops boharti* (Schlinger) comb. n., male, anterior view [700522]. Body length = 9.0 mm.

###### Comments.

*Panops boharti* comb. n.was described by Schlinger (1959) as the sole species in the genus *Neopanops* but is transferred herein to *Panops*. This Indonesian species is the only non-Australian representative of the genus, and is distinctive based on body colouration and markings, very short mouthparts, eye pilosity and eye shape. Only the male is known.

##### Panops conspicuus

(Brunetti, 1926)

http://species-id.net/wiki/Panops_conspicuus

**Figure 34. F34:**
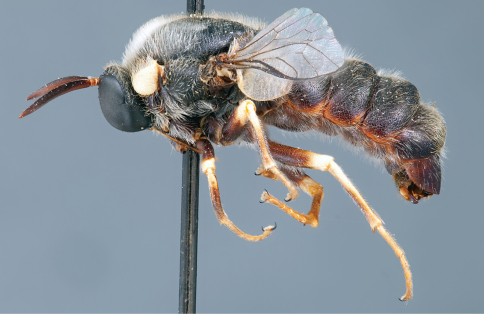
*Panops conspicuus* (Brunetti), male, lateral view [700525]. Body length = 11.0 mm.

Figs 34 [Fig F35] [Fig F36] [Fig F37] –38

Epicerina conspicua Brunetti, 1926: 579.
Panops conspicuus (Brunetti, 1926) – Edwards 1930: 193; Paramonov 1957: 529; Neboiss 1971: 210; Schlinger and Jefferies 1989: 376.

###### Type material examined.

**Holotype** female, AUSTRALIA: **Western Australia:** Kalamunda [-31.974, 116.058], 14.iii–14.iv.1914, R.E. Turner, 1914-349 (BMNH).

###### Other material examined.

AUSTRALIA: **Victoria:** male, female, Kiata [-36.366, 141.791], R. Oldfield, X 4172, captured as copulating pair (NMV). **Western Australia:** female, Boulder Rock [-32.133, 116.166], 15.iii.1981, M.J. Smart, Jarrah Forest, 300m, hovering 2–3 m above ground, taken at rest on leaf (WAM); 4.5 km E Lake Monger on Wanarra Road [-29.544, 116.775], 7.v.2008, T.F. Houston and E. G. Cunningham, 1266-1 (WAM).

###### Diagnosis.

Eye apilose; proboscis longer than head height; body colour and shape sexually dimorphic: male black with slender body, female yellow and brown with globose abdomen; antennae yellow-brown to red-brown with black suffusion; parafacial without marginal pile; postpronotal lobe yellow; legs yellow with brown medially on femora and tibiae.

###### Redescription.

Body length: 11.0 mm (male), 12.0–13.0 mm (female). Head with eye apilose; ocellar tubercle raised laterally; medial ocellus present; occiput colour brown-black (male) or brown with dark yellow spot laterally (female); occipital pile yellow; postocular ridge and gena glabrous; clypeus shorter than oral cavity; yellow-brown; palpus yellow; margin of oral cavity (parafacial) glabrous; proboscis longer than head height; flagellum dark yellow, suffused with brown (female) or red with black suffusion (male), apex in male tapered, narrow apically; scape and pedicel brown. Thorax with postpronotal lobe yellow; scutum black (male) or yellow and brown (markings variable) (female); scutal vestiture dense white pile or dense yellow-gold pile; scutellum black or brown; pleuron brown; coxae brown; femora brown-black, apices dark yellow; tibiae dark yellow or dark yellow, suffused with brown; tarsi dark yellow; lower calypter white, with dark yellow margin; wing hyaline (male) or slightly infuscate (female), venation dark; vein R_4_ with spur vein. Abdomen shape rounded globose, much larger than thorax (female) or cylindrical along length (male), colour orange-yellow or brown-black, vestiture elongate yellow pile (whitish in male).

**Figure 35. F35:**
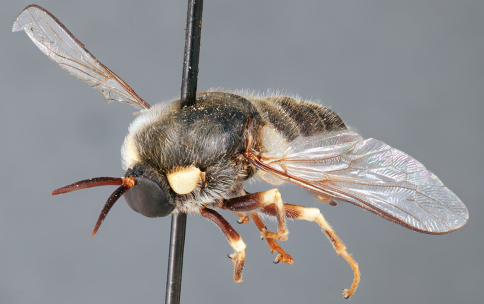
*Panops conspicuus* (Brunetti), male, oblique view [700527]. Body length = 11.0 mm.

**Figure 36. F36:**
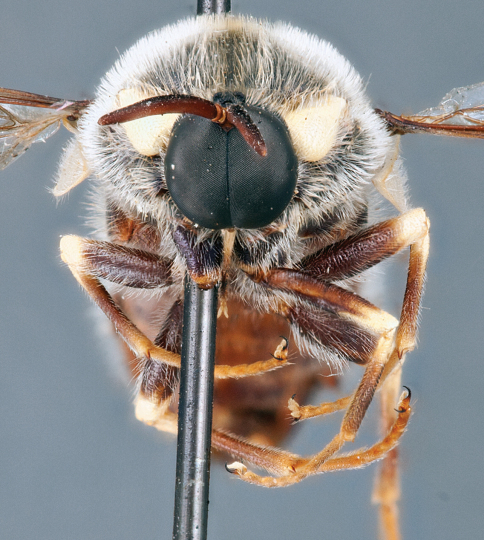
*Panops conspicuus* (Brunetti), male, anterior view [700528]. Body length = 11.0 mm.

**Figure 37. F37:**
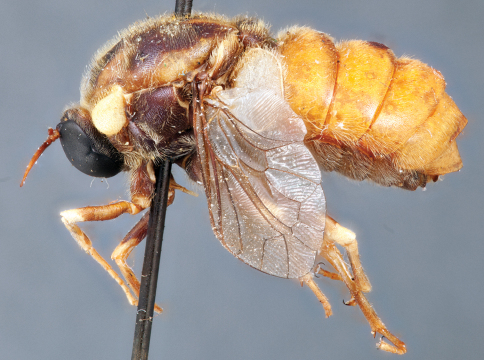
*Panops conspicuus* (Brunetti), female, lateral view [700529]. Body length = 13.0 mm.

**Figure 38. F38:**
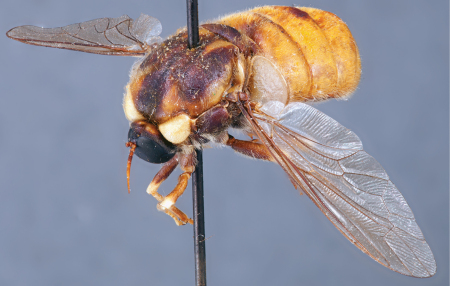
*Panops conspicuus* (Brunetti), female, oblique view [700530]. Body length = 13.0 mm.

###### Comments.

*Panops conspicuus* is recorded from arid regions of southwest Western Australia and Western Victoria. There is dramatic sexual dimorphism in both body colouration and shape in this species, with males very similar to species of *Mesophysa*. *Panops conspicuus* can be differentiated from other *Panops* species by the bright yellow postpronotal lobes, elongate mouthparts, yellow and brown colouration (female), and apilose eyes. Females of this species are similarly coloured to females of *Panops grossi* comb. n., a species which also displays dramatic sexual dimorphism.

##### Panops danielsi sp. n.

urn:lsid:zoobank.org:act:3FAB3406-C6A4-42CC-9ABC-B82BCB22FDE8

http://species-id.net/wiki/Panops_danielsi

**Figure 39. F39:**
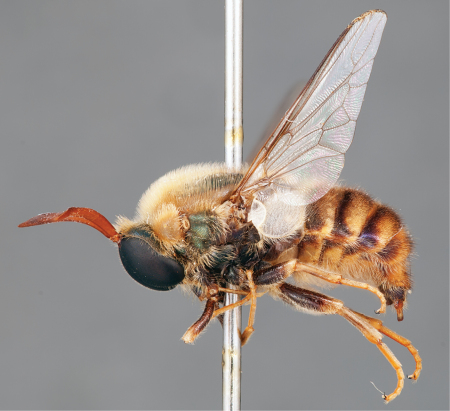
*Panops danielsi* sp. n., male, lateral view [700531]. Body length = 11.0 mm.

Figs 39 [Fig F40] [Fig F41] [Fig F42] –43

###### Type material. 

**Holotype** male, AUSTRALIA: **Queensland:** 3km SW Fox Ck. x-ing [crossing], ‘Wolverton’ [-13.104, 142.970], 13.iv.1989, G. and A. Daniels (AMS).

###### Paratypes.

 AUSTRALIA: **Queensland:** female, male, same data as holotype (GDCB) (CAS); female, 7 km NNW Coen, [-13.844, 143.163], 17.iv.1989, G. and A. Daniels (GDCB); female, 26 km W ‘Fairview’, [-15.535, 144.154], 20.iv.1989, G. and A. Daniels (GDCB).

###### Diagnosis.

Eye uniformly sparse pilose; proboscis longer than head height; body dark yellow and brown, with metallic green-blue iridescence; antennae red-brown or black; parafacial with marginal pile; postpronotal lobe dark yellow; legs dark yellow and brown.

###### Description.

Body length: 11.0 mm (male), 10.5–12.0 mm (female). Head with eye sparsely pilose, uniformly distributed, setae minute; ocellar tubercle raised laterally; medial ocellus absent; occiput metallic green-blue; occipital pile yellow; postocular ridge and gena overlain with grey pubescence; flagellum apex in male uniform width, truncated apically, narrower in female, red-brown (male) or black (female); scape and pedicel dark yellow; clypeus length equal to oral cavity, brown-black; palpus yellow; margin of oral cavity (parafacial) pilose; proboscis longer than head height. Thorax with postpronotal lobe yellow; scutum glossy black (with metallic iridescence), dark yellow marginally; scutal vestiture dense yellow-gold pile; scutellum brown, dark yellow medially; pleuron brown with metallic iridescence; coxae black or brown; femora brown-black, apices dark yellow; tibiae dark yellow, suffused with brown; tarsi dark yellow; lower calypter white, with yellow margin; wing hyaline, venation dark; vein R_4_ with spur vein. Abdomen shape rounded globose, much larger than thorax (female) or rounded to conical, not larger than thorax (male), colour black with metallic green iridescence (female) or dark yellow, brown anteriorly on tergites 2–6 (male), vestiture extensive white-silver elongate setae, brown posteromedially on tergites 3–5 (female) or erect dark pile (male).

**Figure 40. F40:**
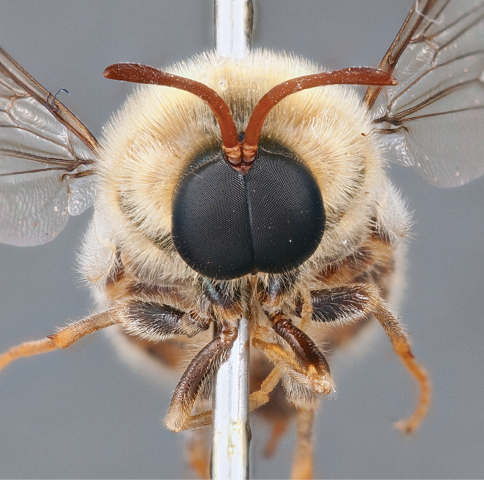
*Panops danielsi* sp. n., male, anterior view [700532]. Body length = 11.0 mm.

**Figure 41. F41:**
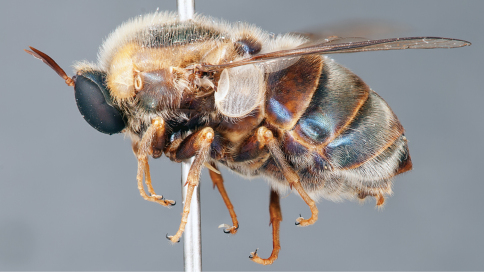
*Panops danielsi* sp. n., female, lateral view [700533]. Body length = 12.0 mm.

**Figure 42. F42:**
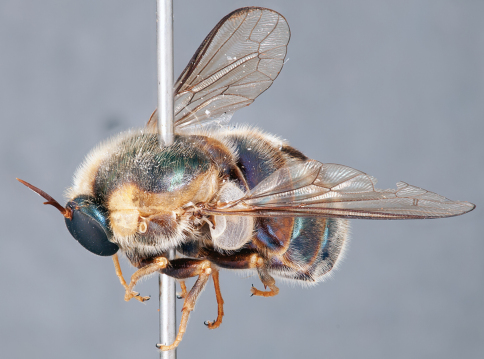
*Panops danielsi* sp. n., female, oblique view [700534]. Body length = 12.0 mm.

**Figure 43. F43:**
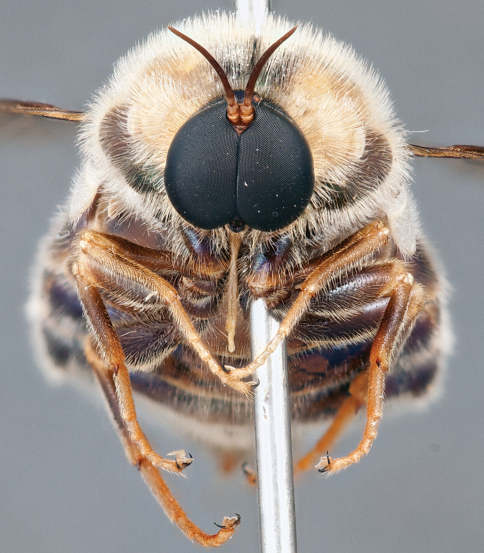
*Panops danielsi* sp. n., female, anterior view [700535]. Body length = 12.0 mm.

###### Etymology.

This species is named in honour of the collector of this species, Greg Daniels.

###### Comments.

*Panops danielsi* sp.n. is known only from Far Northern Queensland. This species is closely related to *Panops baudini* as both species have similar shaped mouthparts and pilose eyes. *Panops danielsi* sp. n. can be distinguished by the more evident eye pilosity, yellow postpronotal lobes and body colouration.

##### Panops grossi

(Neboiss, 1971) comb. n.

http://species-id.net/wiki/Panops_grossi

**Figure 44. F44:**
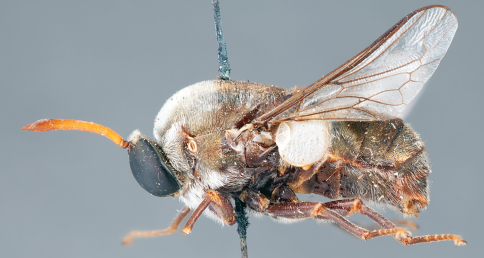
*Panops grossi* (Neboiss) comb. n., male, lateral view [700536]. Body length = 9.0 mm.

Figs 44 [Fig F45] [Fig F46] –47

Panocalda grossi Neboiss, 1971: 214 – Schlinger and Jefferies 1989: 376.

###### Type material examined.

**Holotype** female, AUSTRALIA: **Northern Territory:** Koolpinyah, 21.iv.1916 [-12.331, 131.148] G. F. Hill, (in copula) (SAM).

**‘Allotype’**. AUSTRALIA: **Northern Territory:** same data as holotype (SAM).

###### Diagnosis.

Eye pilose dorsally only, relatively dense and elongate; proboscis shorter than head height; body colour and shape sexually dimorphic: male metallic olive green, female yellow and brown, globose; antennae yellow; parafacial without marginal pile; postpronotal lobe and legs concolourous with rest of body.

###### Redescription.

Body length: 9.0 mm (male), 12.0 mm (female). Head eye pilose dorsally only, dense and relatively elongate; occiput olive green, occipital pile dense white (male) or yellow (female); postocular ridge and gena overlain with grey pubescence; ocellar tubercle raised laterally or relatively flat; medial ocellus absent; clypeus shorter than oral cavity, yellow-brown; palpus black; margin of oral cavity (parafacial) glabrous; proboscis not extending beyond oral cavity; flagellum yellow, apex in male uniform width, truncated apically; scape and pedicel brown. Thorax with postpronotal lobe yellow (female) or green (male); scutum metallic olive green or yellow-orange; scutal vestiture dense white or yellow-gold pile; scutellum metallic olive green or orange-yellow with brown suffusion; pleuron orange or metallic olive green; coxae brown; femora brown-black, apices dark yellow; tibiae brown; tarsi brown; lower calypter white, brown marginally on membrane or white, with dark yellow margin; wing hyaline or slightly infuscate, venation dark; vein R_4_ without spur vein. Abdomen shape with male rounded, not larger than thorax, metallic olive green, vestiture dense short pile, longer laterally; female rounded globose, much larger than thorax (female), orange-yellow (female), vestiture elongate yellow pile.

**Figure 45. F45:**
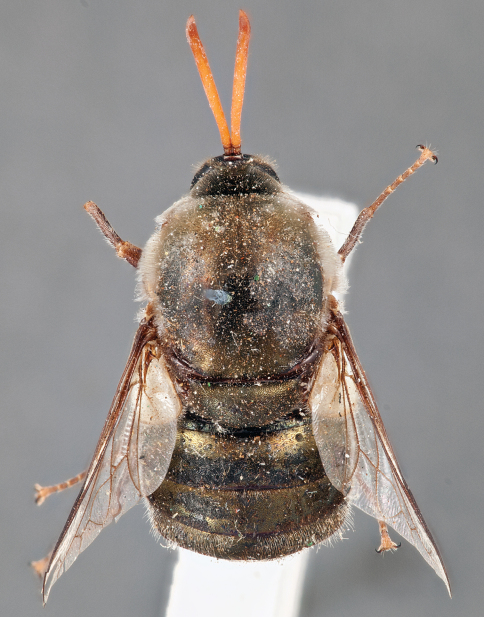
*Panops grossi* (Neboiss) comb. n., male, dorsal view [700537]. Body length = 9.0 mm.

**Figure 46. F46:**
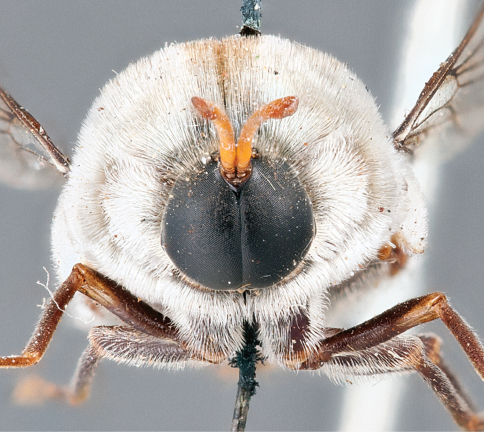
*Panops grossi* (Neboiss) comb. n., male, anterior view [700538]. Body length = 9.0 mm.

**Figure 47. F47:**
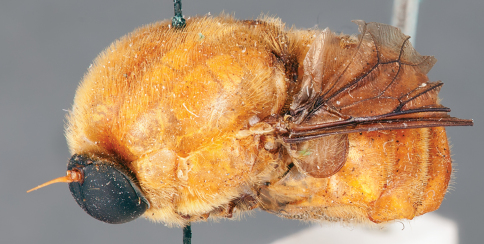
*Panops grossi* (Neboiss) comb. n., female, oblique view [700539]. Body length = 12.0 mm.

###### Comments.

*Panops grossi* comb. n.was described by Neboiss (1971) as the sole species in the genus *Panocalda* but is transferred herein to *Panops*. This species is apparently closely related to *Panops boharti* comb. n. based on eye pilosity, and *Panops schlingeri* sp. n. and *Panops jade* sp. n. based on the short mouthparts. All of these species are northern Australian or Indonesian species. *Panops grossi* comb. n. can be distinguished from all other *Panops* based on the dense patch of relatively elongate pile on the dorsal part of the eye. This species displays a dramatic sexual dimorphism similar to that found in *Panops conspicuus*, with females being orange-yellow in colour.

##### Panops jade sp. n.

urn:lsid:zoobank.org:act:96D0BD2A-0C81-4BCE-BB32-671D1C2D901C

http://species-id.net/wiki/Panops_jade

Figs 2D 48 [Fig F49] [Fig F50] [Fig F51] –52

###### Type material. 

**Holotype** male, AUSTRALIA: **Queensland:** Isla Gorge National Park [-25.183, 149.966], 3.x.1991, 320 m, G. Daniels (AMS).

###### Paratypes.

 AUSTRALIA: **Queensland:** female, Isla Gorge National Park [-25.183, 149.966], 3.x.1991, 320 m, G. Daniels (CAS); female, Isla Gorge National Park [-25.183, 149.966], 14.ix.1992, 320 m, G. Daniels (AMS).

###### Diagnosis.

Eye apilose; proboscis shorter than head height; body metallic green-blue to violet iridescence; antennae red-brown; parafacial with marginal pile; postpronotal lobe concolourous with rest of thorax; legs black with metallic blue-violet iridescence.

###### Description.

Body length: 11.5 mm (male), 11.5–12.0 mm (female). Head with eye apilose; ocellar tubercle relatively flat; medial ocellus present; occiput metallic green-blue, occipital pile white, sparse; postocular ridge and gena overlain with grey pubescence; clypeus length equal to oral cavity, black with blue-green suffusion; palpus black; margin of oral cavity (parafacial) pilose; proboscis extending beyond oral cavity, but shorter than head height; flagellum apex in male tapered, slightly rounded apically, red-brown; scape and pedicel red-brown. Thorax with postpronotal lobe blue-violet; scutum metallic blue-violet, green posteromedially; scutellum metallic blue-violet; coxae and femora with metallic blue-violet iridescence; tibiae black; tarsi black; lower calypter white with brown margin; wing hyaline, venation dark; vein R_4_ with spur vein. Abdomen shape rounded globose, much larger than thorax, colour metallic green or blue-violet iridescent, vestiture extensive white-silver short pile, longer laterally.

**Figure 48. F48:**
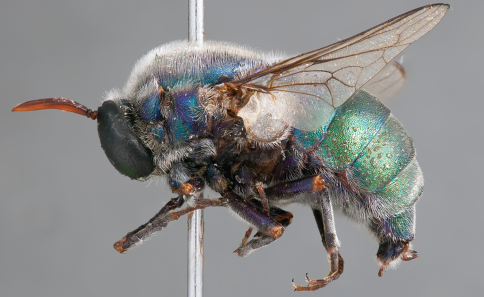
*Panops jade* sp. n., male, lateral view [700540]. Body length = 11.5 mm.

**Figure 49. F49:**
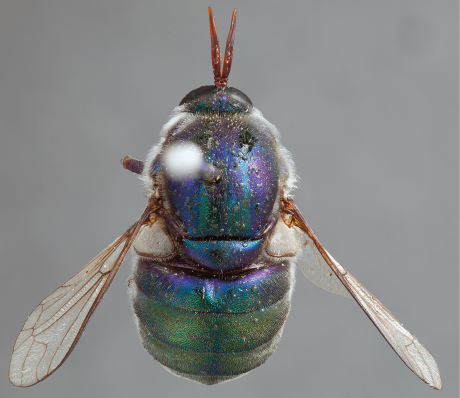
*Panops jade* sp. n., male, dorsal view [700541]. Body length = 11.5 mm.

**Figure 50. F50:**
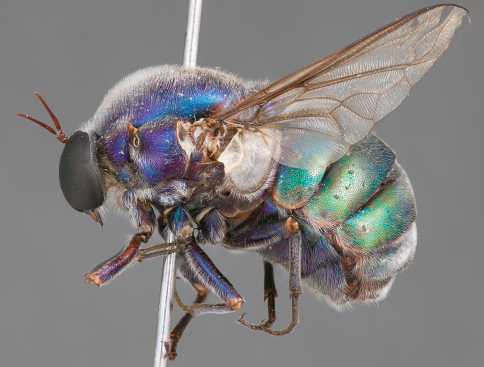
*Panops jade* sp. n., female, lateral view [700542]. Body length = 12.0 mm.

**Figure 51. F51:**
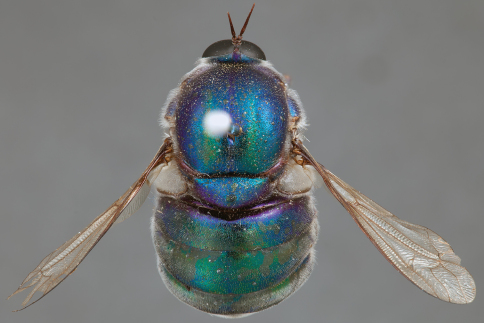
*Panops jade* sp. n., female, dorsal view [700543]. Body length = 12.0 mm.

**Figure 52. F52:**
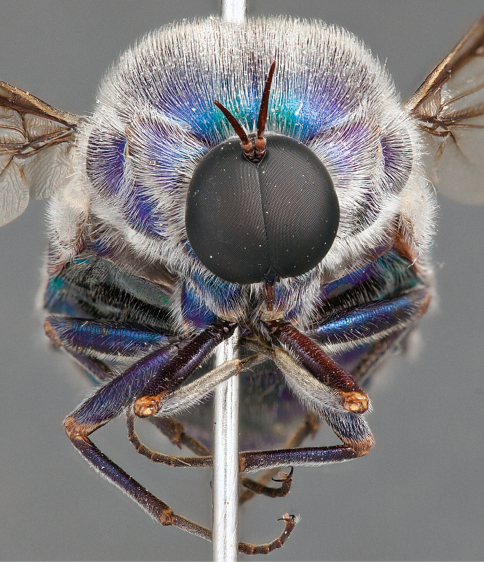
*Panops jade* sp. n., female, anterior view [700545]. Body length = 12.0 mm.

###### Etymology.

This beautifully coloured species is named after my daughter, Jade Tanya Winterton, whose name also describes the deep green colouration found in this species.

###### Comments.

*Panops jade* sp. n. is a distinctive species with extensive green to blue-violet iridescence, particularly in the female. It is similar to the western Australian species, *Panops austrae*, but is distinguished by the length of the mouthparts, leg colour and different vestiture pattern on the abdomen. *Panops jade* sp. n. is known only from Isla Gorge National Park in southern Queensland. Both males and females are recorded from Spinifex grass (*Triodia* sp.), presumably at rest.

##### Panops schlingeri sp. n.

urn:lsid:zoobank.org:act:03D163A1-D1DA-4810-8D88-77F76D5CC490

http://species-id.net/wiki/Panops_schlingeri

**Figure 53. F53:**
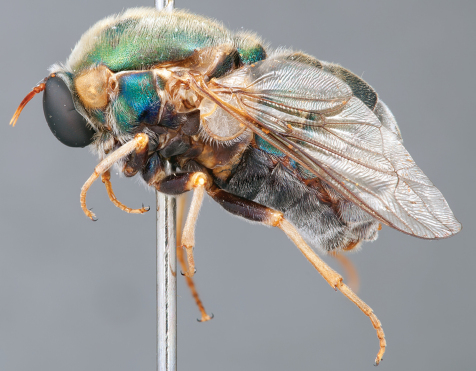
*Panops schlingeri* sp. n., female, lateral view [700546]. Body length = 11.0 mm.

Figs 53 [Fig F54] –55

###### Type material. 

**Holotype** female, AUSTRALIA: **Northern Territory:** 9 km NE of Mudginbarry H.S. (on scarp), 10.vi.1973, D. H. Colless [-12.310, 132.579] (ANIC).

###### Paratype.

 AUSTRALIA: **Northern Territory:** female, 8 km SSW of Oenpelli Mission 7.vi.1973, J. Cardale [-12.381, 133.024] (ANIC).

###### Diagnosis.

Eye apilose; proboscis shorter than head height; body metallic green-blue iridescence; antennae orange; parafacial without marginal pile; postpronotal lobe dark yellow; legs dark yellow, femora brown-black with yellow apices.

###### Description.

Body length: 9.5–11.0 mm (female only). Head with eye apilose; ocellar tubercle relatively flat; medial ocellus present; occiput metallic green-blue, occipital pile white, dense; postocular ridge and gena overlain with grey pubescence; clypeus shorter than oral cavity, brown-black; palpus black; margin of oral cavity (parafacial) glabrous; proboscis not extending beyond oral cavity; flagellum orange; scape and pedicel dark red-yellow. Thorax with postpronotal lobe yellow; scutum metallic green to blue iridescent; scutal vestiture dense white pile; scutellum metallic blue-green; pleuron metallic green to blue iridescent; coxae brown-black with metallic blue iridescence; femora brown-black, apices dark yellow; tibiae dark yellow; tarsi dark yellow; lower calypter white, with dark yellow margin; wing hyaline, venation dark; vein R_4_ without spur vein. Abdomen shape rounded globose, much larger than thorax, dark with metallic green to blue iridescence, vestiture as dense short pile, longer laterally.

**Figure 54. F54:**
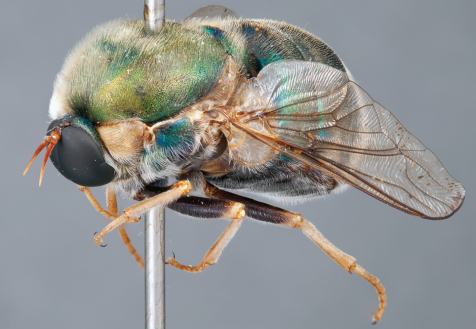
*Panops schlingeri* sp. n., female, oblique view [700547]. Body length = 11.0 mm.

**Figure 55.**
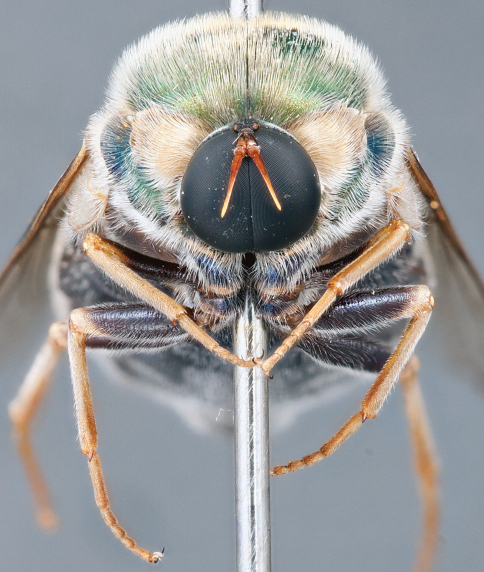
*Panops schlingeri* sp. n., female, anterior view [700548]. Body length = 11.0 mm.

###### Etymology.

I am honoured to name this species after the world-renowned Acroceridae taxonomist Dr. Evert Irving Schlinger.

###### Comments.

*Panops schlingeri* sp. n. is known only from two female specimens collected in the Northern Territory. This species is differentiated easily by the green-blue iridescence on the body and dark yellow postpronotal lobes.

##### Subfamily Philopotinae

Schiner, 1968

http://species-id.net/wiki/Philopotinae

###### Type genus.

*Philopota* Wiedemann. Schlinger, 1971: 186.

###### Diagnosis.

Body shape slightly to strongly arched and never densely pilose; small to medium sized; antennal flagellum stylate; postpronotal lobes enlarged and meeting medially to form collar behind head; tibial spines absent; wing costal vein ending at wing apex, never circumambient; wing venation highly variable, ranging from relatively complete with cells cu-p, bm br, d and basal r_4+5 _present, to highly reduced with only cell br present; cell m_3_ absent; veins R_4 _and R_5_ always present as single vein R_4+5_; cubital and medial veins not reaching posterior wing margin; larvae exclusively parasitoids of araneomorph spiders.

### Australasian genera

*Helle* Osten Sacken, 1896; *Schlingeriella* Gillung & Winterton, 2011.

#### Helle

Osten Sacken, 1896

http://species-id.net/wiki/Helle

Figs 3A 56 [Fig F57] [Fig F58] –59

Helle Osten Sacken, 1896: 16 – Hutton 1901: 28; Paramonov 1955: 21; Schlinger and Jefferies 1989: 376. Type species: *Acrocera longirostris* Hudson, 1892: 56 by monotypy.

##### Diagnosis.

 Body length: 4.0–6.0 mm [male], 6.0–7.0 mm [female]. Body shape strongly arched; colouration non-metallic (brown or black); head size slightly narrower than thorax width, shape sub-spherical; postocular ridge and occiput rounded; three ocelli, anterior ocellus reduced in size; posterior margin of eye rounded; eye apilose; position of antennae on head near middle of frons; eyes contiguous above antennal base, not contiguous below antennal base; palpus present; proboscis greater than head length; flagellum stylate, apex with terminal seta; postpronotal lobes enlarged, medially contiguous to form collar; subscutellum enlarged; legs not elongated; wing markings absent; costa ending near wing apex, costal margin straight; humeral crossvein absent; radial veins straight or curved towards wing anterior margin; R_1_ inflated distally at pterostigma; pterostigma and cell r_1_ membranous, not ribbed; R_2+3 _present; R_4+5 _angled anteriorly approximately midway; cell r_4+5 _bisected by 2r-m, basal cell very narrow elongate, closed; 2r-m joining M_1_ to R_4+5_; cell r_4+5 _present, narrow elongate, closed (open apically when 2r-m rarely absent); crossvein 2r-m present (rarely absent);R_4_ without spur vein; medial vein compliment with M_1_, M_2_ and M_3_ present (M_3_ fused with CuA_1_); discal cell closed completely; medial veins not reaching wing margin; CuA_1_ joining M_3_, petiolate to margin; CuA_2_ fused to A_1_ before wing margin, petiolate; wing microtrichia absent; anal lobe well developed; alula well developed; abdominal tergites smooth, rounded; abdomen shape elongate, narrow cylindrical or conical (male), or rounded and inflated (female).

**Figure 56.**
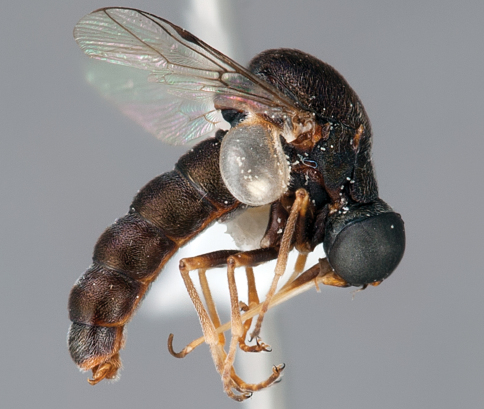
*Helle longirostris* (Hudson), male, lateral view [700556]. Body length = 5.0 mm.

**Figure 57. F57:**
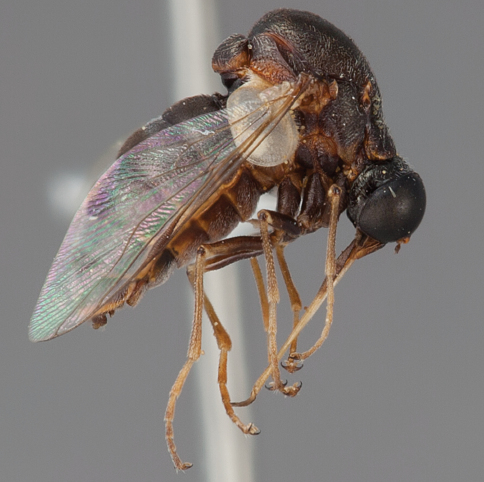
*Helle longirostris* (Hudson), female, lateral view [700557]. Body length = 5.5 mm.

**Figure 58. F58:**
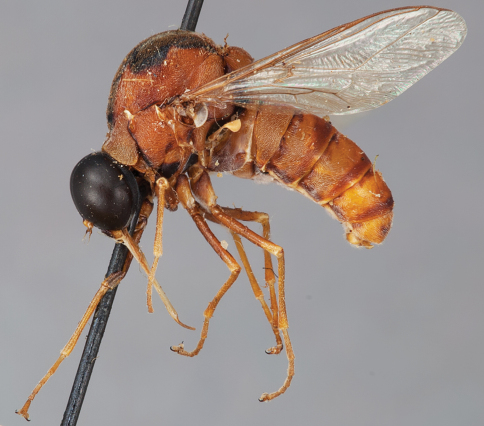
*Helle rufescens* Brunetti, male, lateral view [700558]. Body length = 8.5 mm.

**Figure 59.**
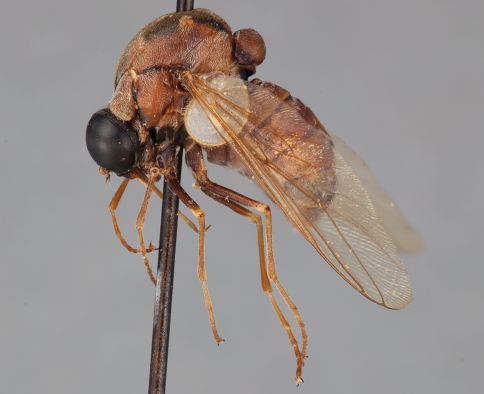
*Helle rufescens* Brunetti, female, lateral view [700559]. Body length = 7.0 mm.

##### Included species.

*Helle longirostris* (Hudson, 1892); *Helle rufescens* Brunetti, 1926.

##### Comments.

*Helle* is an endemic genus to New Zealand that is closely related to *Schlingeriella*, the only other philopotine genus in the region (Gillung and Winterton 2011; Winterton et al. 2007). Characteristics supporting this closerelationship include thickening of wing vein R_1_ at the pterostigma, elongate mouthparts, apilose eyes, 2r-m absent (rarely in *Helle*) and R_4+5 _angled anteriorly approximately half way along vein. *Helle* can be differentiated from all other philopotine genera based on the relatively complete wing venation, inflated R_1_ at pterostigma, palpi present and apilose eyes.

##### Key to Helle species

**Table:** 

1	Body colour brown-black, sometimes with metallic iridescence, scutum without dark markings (Figs 56–57)	*Helle longirostris* (Hudson, 1892)
–	Body colour yellowish-orange, scutum with dark longitudinal stripes, narrower anteriorly (Figs 58–59)	*Helle rufescens* Brunetti, 1926

#### Schlingeriella

Gillung & Winterton

urn:lsid:zoobank.org:act:99EAC1BE-4A6F-43E0-B61A-6460BF68694E

http://species-id.net/wiki/Schlingeriella

Figs 3B 60 [Fig F61] –62

Schlingeriella Gillung & Winterton, 2011: 22. Type species: *Schlingeriella irwini* Gillung & Winterton, 2011: 23.

##### Diagnosis.

Body length: 2.4–4.0 mm [male], 4.4–6.0 mm [female]. Body shape arched; body colouration non-metallic dark brown; head width much smaller than thorax (female) or slightly smaller than thorax (male); head spherical; postocular ridge and occiput extended posteriorly into slight ridge; posterior margin of eye rounded; eyes bare; position of antennae on head near middle of frons, slightly nearer to mouthparts; eyes contiguous above antennal base, not contiguous below; palpus present; proboscis longer than head; antennal flagellum stylate, apex with terminal seta; thorax with postpronotal lobes enlarged, medially contiguous to form collar; subscutellum enlarged; legs not greatly elongated; pulvilli present; wing hyaline, markings absent; costa ending in radial field; costal margin straight in both sexes; humeral crossvein absent; radial veins meeting wing margin before wing apex; R_1_ inflated distally at pterostigma; R_2+3 _present; R_4+5 _slightly curved anteriorly midway; veins M_1_, M_2_ and M_3_ present; discal cell absent; medial veins reaching wing margin (or nearly so); crossvein 2r-m absent; Cu reduced, not reaching wing margin; anal lobe not enlarged; alula well developed; abdomen smooth, rounded, cylindrical in shape, similar width to thorax (male) or greatly rounded, inflated (female).

**Figure 60.**
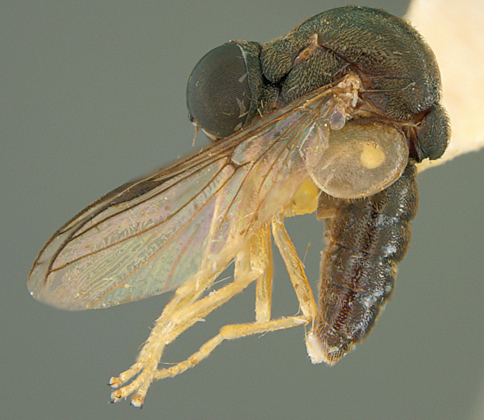
*Schlingeriella irwini* Gillung & Winterton, male, lateral view [700560, 693079]. Body length = 2.4 mm.

**Figure 61. F61:**
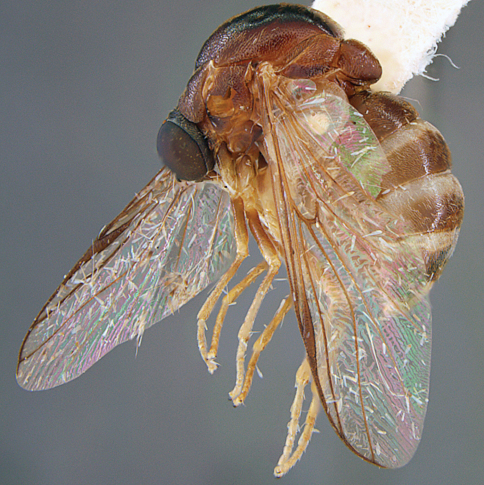
*Schlingeriella irwini* Gillung & Winterton, female, lateral view [700561, 693080]. Body length = 4.4 mm.

**Figure 62.**
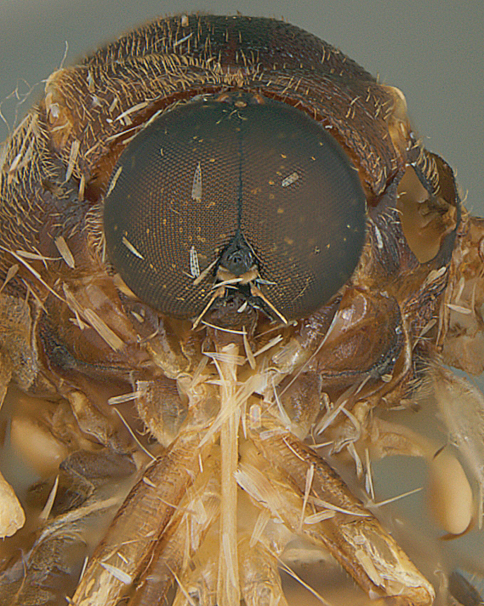
*Schlingeriella irwini* Gillung & Winterton, female, anterior view [700562]. Body length = 4.4 mm.

##### Included species.

*Schlingeriella irwini* Gillung & Winterton, 2011.

##### Comments.

*Schlingeriella* is differentiated from other Philopotinae by medial veins mostly reaching the wing margin, R_1_ inflated apically, reduced wing venation (i.e. absence of all wing cells except cell *br*), elongate mouthparts and apilose eyes. See results of Winterton et al. (2007) for phylogenetic placement and divergence times. This genus is represented by only a single species (*Schlingeriella irwini* sp. n.) from New Caledonia (France). There is dramatic sexual dimorphism in body size, with females considerably larger than the males. This genus was described by Gillung and Winterton (2011) to honour the decades of work by Evert I. Schlinger on world Acroceridae taxonomy. Evert Schlinger not only collected many of the specimens in New Caledonia, he also recognized that it represented a completely new genus of endemic spider flies.

#### Subfamily Acrocerinae

Zetterstedt, 1837

http://species-id.net/wiki/Acrocerinae

##### Type genus.

 Acrocera Meigen 1803: 266.

##### Diagnosis.

Small to medium sized, densely pilose to apilose, body rarely arched; antennal flagellum stylate; postpronotal lobes widely separated, never medially contiguous; wing venation highly variable, ranging from complete with cells cu-p, bm br, d, m_3_ and basal r_4+5 _present, to highly reduced with few closed cells; humeral crossvein rarely well developed; tibial apical spines absent (rarely present); larvae exclusively parasitoids of araneomorph spiders.

### Australasian genera

*Ogcodes* Latreille, 1797; *Pterodontia* Gray, 1832

#### Ogcodes

Latreille, 1797

http://species-id.net/wiki/Ogcodes

Figs 3C 63 –64

Ogcodes Latreille, 1797: 154 – Schlinger 1960: 245; Schlinger and Jefferies 1989: 377. Type species, *Musca gibbosa* Linnaeus, by subsequent monotypy (Latreille 1802: 432).
Oncodes Meigen 1822: 99 [emendation of *Ogcodes* Latreille] – White 1914: 69; Hardy 1921: 77, 1946: 66; Paramonov 1955: 23, 1957: 531.

##### Note.

 Synonymy and usage restricted to Australasian region fauna only; see Schlinger (1960) for more exhaustive list.

##### Diagnosis.

 Body length: 3.0–5.0 mm [male], 4.0–8.0 mm [female]. Body shape not arched, colouration black, yellow or white, non-metallic; head much smaller than thorax width, shape sub-spherical; postocular ridge and occiput rounded; two or three ocelli, anterior ocellus sometimes absent; posterior margin of eye rounded; eye apilose; position of antennae on head adjacent to mouthparts; eyes contiguous above antennal base, not contiguous below antennal base; palpus absent; proboscis apparently absent; flagellum shape stylate; apex with terminal setae (or single seta); antenotum not collar-like behind head; subscutellum enlarged; tibial spines absent; pulvilli present; wing hyaline, markings absent; costa ending near wing apex, costal margin straight; humeral crossvein absent; radial veins straight; R_1_ inflated or not inflated distally; pterostigma and cell r_1_ membranous, not ribbed; only two radial veins present, R_2+3 _absent, R_4+5 _not reaching wing margin; medial vein compliment with M_1_, M_2 _and M_3_ present, or two M veins present; discal cell weakly formed or absent; medial veins not reaching wing margin; cell m_3_ absent; CuA_1_ absent; CuA_2_ separate from A_1_, ending just before wing margin; crossvein 2r-m absent; wing microtrichia absent; anal lobe well developed; alula well developed; abdominal tergites smooth, rounded (rarely with tubercles in fossil species); abdomen shape greatly rounded, inflated.

**Figure 63.**
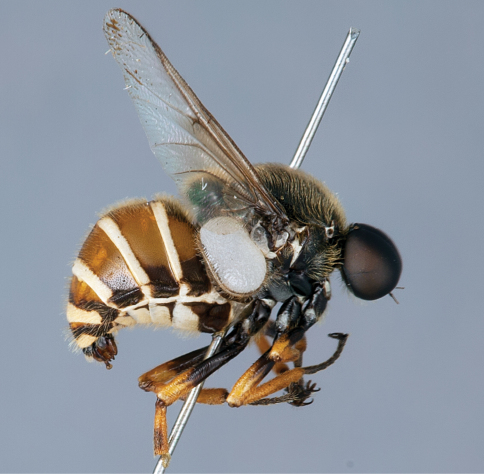
*Ogcodes* sp., male, lateral view [700563]. Body length = 9.0 mm.

**Figure 64.**
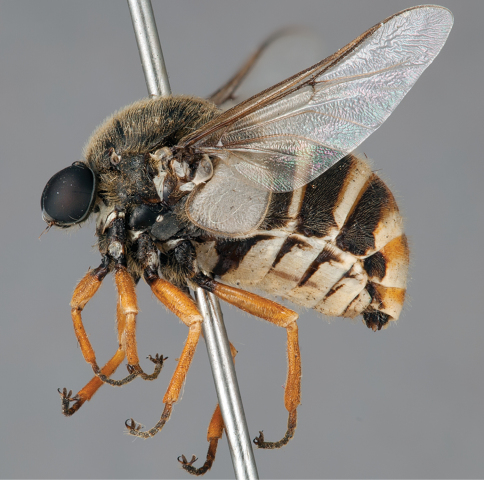
*Ogcodes* sp., female, lateral view [700564]. Body length = 5.0 mm.

##### Included species.

*Ogcodes* is a distinctive and cosmopolitan genus and the most species-rich in the family. Thirty-four species in two subgenera (*Ogcodes* and *Protogcodes* Schlinger, 1960) are listed by Schlinger and Jefferies (1989) for the Australasian region.

##### Comments.

*Ogcodes* is in need of revision and no recent keys to species have been published for the region. The most recent revision of the genus was by Schlinger (1960), but there are many undescribed species in collections and a world revision of the genus is needed. *Ogcodes* is a derived genus with a typical globose body, relatively small head and reduced wing venation. Characters which differentiate *Ogcodes* from all other Acroceridae genera include antennae proximal to mouthparts, palpi absent, proboscis very short, almost all wing cells absent or poorly formed, eyes apilose and R_2+3 _absent.

#### Pterodontia

Gray, 1832

http://species-id.net/wiki/Pterodontia

Figs 3D 65 –66

Pterodontia Gray, 1832: 779 – Macquart 1838: 174; Erichson 1840: 161; Walker 1855: 346; Westwood 1876: 513; White 1914: 68; Hardy 1921: 76, 1946: 66; Paramonov 1957: 529; Schlinger 1959: 158. Type species: *Pterodontia flavipes* Gray, 1832: 779 by monotypy.
Nothra Westwood, 1876: 514 – Hardy 1921: 77, 1946: 66. Type species: *Nothra bicolor* Westwood, 1876: 514 by monotypy.

##### Note.

 Synonymy and usage list restricted to Australasian region fauna only.

##### Diagnosis.

Body length: 3.0–7.0 mm [male], 4.0–10.0 mm [female]. Body shape not arched. Body colouration non-metallic; head much narrower than thorax width; shape nearly spherical; postocular ridge and occiput rounded; three ocelli; posterior margin of eye rounded; eye pilose (dense); antennae located adjacent to mouthparts; eyes contiguous above antennal base, not contiguous below antennal base; palpus absent; proboscis greatly reduced; flagellum stylate, apex with terminal setae (multiple); antenotum shape not collar-like behind head; subscutellum not enlarged, barely visible; tibial spines present; pulvilli present; wing markings absent; costa circumambient; wing costal margin straight or with anterior projection (males); humeral crossvein present or reduced; radial veins curved or angled towards wing anterior margin; R_1_ inflated distally at pterostigma (especially in male); pterostigma and cell r_1_ membranous, not ribbed; R_2+3 _present; R_4+5 _present as single vein; basal cell r_4+5 _(portion basal to bisecting 2r-m) merged with discal cell to form composite cell comprising d+r_4+5_; cell m_3_ absent; medial vein compliment usually a single M vein fused with CuA_1_, petiolate to margin, sometimes with second medial vein originating from cell d+r_4+5_; CuA_2_ fused to A_1_ before wing margin, petiolate, rarely open to wing margin; wing microtrichia absent; anal lobe well developed; alula present or absent, rarely well developed; abdominal tergites smooth, rounded; abdomen shape greatly rounded, inflated.

**Figure 65.**
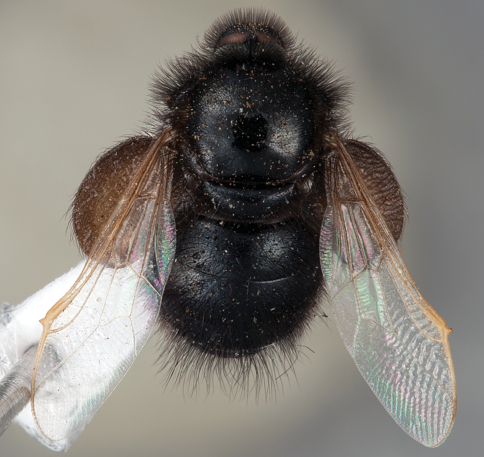
*Pterodontia davisi* Paramonov, male, dorsal view [700565]. Body length = 7.0 mm.

**Figure 66.**
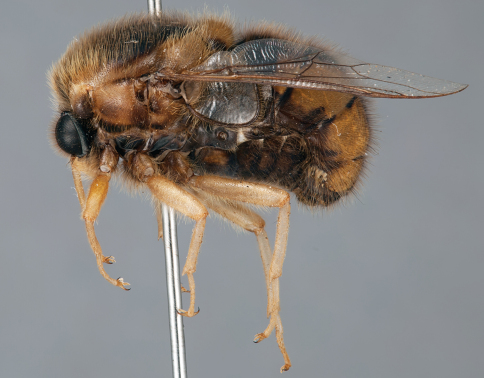
*Pterodontia mellii* Erichson, female, lateral view [700566]. Body length = 11.0 mm.

##### Included species.

*Pterodontia davisi* Paramonov, 1957; *Pterodontia longisquama* Sabrosky, 1947; *Pterodontia mellii* Erichson 1840 (= *Pterodontia variegata* White, 1914 syn. n.).

##### Key to Australasian Pterodontia species

**Table:** 

1	Thorax black, or yellow suffused with dark brown to black ventrally,abdomen yellow to red laterally on segments 2–4; mid and hind femora brown to black; lower calypter hyaline medially, relatively small (< ½ length of wing) (Western Australia, Tasmania, New South Wales, Queensland) (Fig. 66)	*Pterodontia melli* Erichson 1840
–	Thorax and abdomen completely brown to black; all legs uniformly yellow to white; lower calypter relatively large (> ½ length of wing), uniformly brown	2
2	Wing brown infuscate (Papua New Guinea)	*Pterodontia longisquama* Sabrosky, 1947
–	Wing hyaline (Queensland, New South Wales) (Fig. 65)	*Pterodontia davisi* Paramonov 1957

##### Comments.

*Pterodontia* is a cosmopolitan genus containing 19 valid species, three of which are recorded from the Australasian region (Schlinger and Jefferies 1989). *Pterodontia variegata* was described by White (1914) and differentiated from *Pterodontia melli* (as *Pterodontia macquarti* Westwood, 1848) based on colouration of the fore femur, scutellum and abdomen. Paramonov (1957) examined a range of specimens from various localities and suggested that the former was likely a synonym of the latter. Based on examinations of these and additional specimens this synonymy is supported herein.

Some species of *Pterodontia* have greatly enlarged and sclerotized lower calypters, appearing somewhat like a second pair of wings (e.g. *Pterodontia davisi*). Males in this genus typically have sclerotized projections on the costal margin of the wing. Characteristics which diagnose this genus from other acrocerids include head very small relative to thorax width, tibial spines present, cells m_3_, d and basal r_4+5 _fused to form a single cell, eyes densely pilose, antennae adjacent to the ocellar tubercle and mouthparts reduced. Contrary to other authors, *Pterodontia* has been placed previously in Panopinae by Schlinger (1981, 1987, 1989) based on the presence of tibial spines. The wing venation of *Pterodontia* is unique among acrocerids.

## Supplementary Material

XML Treatment for Panopinae

XML Treatment for Apsona

XML Treatment for Leucopsina

XML Treatment for Mesophysa

XML Treatment for Panops

XML Treatment for Helle

XML Treatment for Schlingeriella

XML Treatment for Acrocerinae

XML Treatment for Ogcodes

XML Treatment for Pterodontia
